# Potent efficiency of the novel nitazoxanide-loaded nanostructured lipid carriers against experimental cyclosporiasis

**DOI:** 10.1371/journal.pntd.0011845

**Published:** 2023-12-15

**Authors:** Nancy Abd-elkader Hagras, Shaimaa Makled, Eman Sheta, Mohamed Ali El-hawary, Nermine Mogahed Fawzy Hussein Mogahed

**Affiliations:** 1 Department of Medical Laboratory Technology, Faculty of Applied Health Sciences Technology, Pharos University in Alexandria, Alexandria, Egypt; 2 Department of Pharmaceutics, Faculty of Pharmacy, Alexandria University, Alexandria, Egypt; 3 Department of Pathology, Faculty of Medicine, Alexandria University, Alexandria, Egypt; 4 Faculty of Science, Zagazig University, Zagazig, Egypt; 5 Department of Medical Parasitology, Faculty of Medicine, Alexandria University, Alexandria, Egypt; University of North Carolina at Chapel Hill, UNITED STATES

## Abstract

Cyclosporiasis is a ubiquitous infection caused by an obligate intracellular protozoan parasite known as *Cyclospora cayetanensis* (*C*. *cayetanensis*). The disease is characterized by severe diarrhea which may be regrettably fatal in immunosuppressed patients. The commercially available treatment options have either severe side effects or low efficiency. In the present study, the novel formula of nitazoxanide (NTZ)-loaded nanostructured lipid carriers (NLCs) was assessed for the first time for *C*. *cayetanensis* treatment in both immunocompetent and immunosuppressed mice in comparison to commercially available drugs (trimethoprim-sulfamethoxazole (TMP-SMX) and NTZ). Swiss Albino mice were orally infected by 10^4^ sporulated oocysts. The experimental groups were treated with the gold standard TMP-SMX, NTZ, blank NLCs and NTZ-loaded NLCs. The results demonstrated that NTZ-loaded NLCs represented the highest significant parasite percent reduction of (>98% reduction) in both immunocompetent and immunosuppressed mice designating successful tissue penetration and avoiding recurrence of infection at the end of the study. Oocysts treated with NTZ-loaded NLCs demonstrated the most mutilated rapturing morphology via scanning electron microscope examination as well as representing the most profound improvement of the histopathological picture. In conclusion, NTZ-loaded NLCs exhibited the uppermost efficacy in the treatment of cyclosporiasis. The safe nature and the anti-parasitic effect of the novel formulation encourage its use as a powerful treatment for human cyclosporiasis.

## Author summary

Cyclosporiasis is an intestinal disease that results from infection with a single-celled parasite known as *Cyclospora cayetanensis* (*C*. *cayetanensis*). Multiple global outbreaks are associated with the parasite. Risk of infection rises with people traveling or living in the endemic countries. Infection occurs frequently by consuming food or water contaminated with *C*. *cayetanensis*. The disease is mainly manifested by severe watery diarrhea which may relapse several times. People with weakened immune systems are at higher risk of severe illness that may unfortunately end up with death. There are several treatment options, however they have severe undesirable side effects. Nitazoxanide is the safest treatment alternative but has a comparable lower efficacy. Consequently, the present work focused on reaching a better therapeutic outcome through the preparation of a novel formula at the nano size range. The novel formulation was achieved by preparing the safe organic nano-structured lipid carriers and loading them with nitazoxanide. The novel formula showed a remarkable potency compared to the commercially available drugs. Subsequently, a new auspicious vista is introduced for cyclosporiasis treatment.

## Introduction

*Cyclospora cayetanensis* (*C*. *cayetanensis*) is an emerging protozoan parasite recognized as an obvious global cause of intestinal illness [1]. The parasite has been documented in developing and developed countries as well. The developing endemic countries extend to several regions including Central and South America, South East Asia, the Indian subcontinent and multiple countries in North Africa and the Middle East. Additionally, hefty outbreaks of cyclosporiasis have grabbed the attention in developed countries as United States, United Kingdom, Canada and Germany. Cyclosporiasis is considered to be one the most common causes of traveler’s diarrhea as visitors to endemic areas are more susceptible to the symptomatic disease than the native population. The disease prevalence widely varies among countries reaching up to 41.6% [1–4].

Man plays the role of the exclusive natural host for the parasite. Human infection occurs through the ingestion of sporulated oocysts in contaminated food and water [2]. The disease is characterized by the high potentiality of recurrence along with causing substantial morbidity and mortality in both immunocompetent and immunocompromised individuals [5]. In immunocompetent patients, the disease is manifested by explosive, watery and foul-smelling diarrhea accompanied with abdominal pain, nausea, vomiting and weight loss [6]. However, lethality may be a prevalent outcome in immunocompromised individuals. Besides, extraintestinal complications as acalculous cholecystitis and thickened gallbladder have been documented [3,6].

Chemotherapeutic treatment is vital in order to conquer cyclosporiasis, particularly in immunocompromised patients. The first line of therapy is trimethoprim-sulfamethoxazole (TMP-SMX) which blocks two successive steps in the synthesis of the parasitic nucleic acids [2,7,8]. Despite its efficacy, recurrence may arise in more than 50% of the patients within one to three months. Additionally, gastrointestinal, renal and hematologic adverse effects are apparently associated with its use [9]. The commonly known sulfa allergy may additionally abandon its use as well [10]. Ciprofloxacin is another treatment option for patients who exhibit intolerance or allergy to TMP-SMX. However, it possesses less effectiveness than the first line therapy [3,11]. Nitazoxanide (NTZ) is another treatment alternative that can be used in the cases of TMP-SMX intolerance and ciprofloxacin resistance. Although NTZ has lower efficacy against cyclosporiasis due to its low solubility, yet its tolerance level is found to be considerably high without having obvious side effects. Thus, there is a crucial need for further development of a nitazoxanide vehicle in order to benefit from its marvelous advantages [1,12].

Nanoscience has recently gained popularity due to its success in treating different diseases. It has the potential capability of improving the drug formulation. Nanoparticles ensure the drug delivery to all body tissues as it enhance the drug bioavailability and permeability and hence lower the drug doses required [11,13].

Lipid-based nanocarriers have offered a new frontier in the treatment of various diseases as several formulations are approved by the Food and Drugs Administration (FDA) [14,15]. They have obtained prodigious interest due to their safe, biodegradable and biocompatible nature [15,16]. Additionally, lipid nanocarriers can evidently boost the drug solubility and pharmacokinetics, which in turn aid in bypassing the biological barriers and decreasing the drug adverse effects. They are favored over polymeric nanoparticles as they can be produced in large-scale [16]. There are several forms of lipid nanocarriers as liposomes, niosomes, solid lipid nanoparticles and nanostructured lipid carriers (NLCs) [17]. NLCs are grabbing attention over the other lipid carriers as they can substantially improve the drug stability, loading capacity and avoid the drug expulsion during storage [18].

In the present study, the therapeutic efficacy of TMP-SMX, NTZ, blank NLCs and the novel NTZ-loaded NLCs has been assessed in immunocompetent and immunosuppressed murine model infected with *C*. *cayetanensis*.

## Material and methods

### I. Ethics statement

The protocol of the present experimental study was approved by the Ethics Committee of the Faculty of Medicine, Alexandria University (protocol approval number: 0306104). The experiments on animals were performed according to Faculty of Medicine institutional considerations for ethical care and use of animals following the guidelines of ICLAS (International Council for Laboratory Animal Science).

### II. Parasite

*C*. *cayetanensis* oocysts were isolated from diarrheic stool sample of HIV positive patient at Main Alexandria University Hospital after taking the verbal informed consent. A metal mesh was used to sieve the fecal suspension in order to remove debris, followed by centrifugation at 2000 rpm for 12 min. The sample was then stained by modified Ziehl Neelsen and safranin stains to specifically ensure *Cyclospora* infection. In order to confirm the diagnosis, the size of the observed oocyst was measured by the ocular micrometer of a light microscope to exclude *Cryptosporidium* oocysts. The isolated oocysts were stored in 2.5% aqueous potassium dichromate at 4 °C to keep them alive for further research use [9,19,20].

### III. Artificial sporulation of oocysts

Preserved *Cyclospora* oocysts samples were allowed to sporulate in covered Petri dishes at room temperature (22–30 °C). Daily microscopic observation of oocysts was done until sporulation occurred within a period of 8–14 days. Sporulated oocysts were furtherly used in induction of murine model of cyclosporiasis [21].

### IV. Drugs

The following drugs were used in the present study:

- Cyclophosphamide (Endoxan vial, Baxter pharmaceutical company, USA) was used to induce a state of immunosuppression in mice.
- The gold standard trimethoprim-sulfamethoxazole (TMP-SMX) (Sutrim suspension, Aspen pharmaceutical company, Germany).
- Nitazoxanide (NTZ) (Nanazoxid suspension, Utopia pharmaceutical company, Cairo, Egypt).
- Blank nanostructured lipid carriers (NLCs) (Stearic acid was supplied by Pharco pharmaceutical company, Alexandria, Egypt. Oleic acid was obtained from Nice chemicals, Kerala, India. Lebrafil m 1944 CS was provided by Gattefossé, Saint Priest, France. Poloxamer188 (P188) was purchased from BASF, Ludwigshafen, Germany).
- NTZ-loaded NLCs (NTZ pure powder was kindly provided by Utopia pharmaceutical company, Cairo, Egypt).

#### IV.1. Preparation of nano-formulations (blank NLCs and NTZ-loaded NLCs)

NLCs were formulated through probe ultrasonication method in accordance with Shawky et al. (2022) [22]. Water bath was used to melt certain amounts of stearic acid (fatty acid) with concentration range from 2 to 8%, in addition to oleic acid with concentration range between 0.1–0.5% and Lebrafil m 1944 CS (mono-, di- and triglycerides and PEG-6 mono- and diesters of oleic acid) with concentration range of 0.2–0.8% in the presence or absence of the drug (0.065 M NTZ) at 80 °C. At the same temperature of the molten lipid, 10 milliliters (ml) of previously heat up Poloxamer188 (P188) surfactant solution (0.5 or 1%) was dropwise poured to the molten lipid phase. The previous blend was later ultra-sonicated at a power output of 60% amplitude for 5 min (Sonoplus HD 3100; Bandelin, Berlin, Germany). The obtained product was poured to 10 ml cold water probe-sonicated at the same power output for 5 min while submerged in an ice bath. The influence of various formulation variables: P188 solution concentration and volume as well as lipid to drug ratio was investigated. The optimized formulation composed of 6% stearic acid, 0.6% oleic acid 0.8% Lebrafil and 1% P188. It was kept at 4 °C and recharacterized after 30 days to ensure its stability. Lyophilization was performed for concentration of both NLCs and NTZ-loaded NLCs using Telestar LyoQuest Lyophilizer (Terrassa, Spain) [22].

#### IV.2. In vitro characterization of nano-formulations (blank NLCs and NTZ-loaded NLCs)

*IV*.*2*.*1*. *Colloidal properties and morphology of blank NLCs and NTZ-loaded NLCs*. NLCs and NTZ-loaded NLCs were evaluated at zero time and after 30 days subsequent to proper dilution with deionized water in terms of particle size (PS), polydispersity index (PDI) and Zeta potential (ZP) by dynamic light scattering (DLS) utilizing a Malvern Zetasizer (Zetasizer Nano ZS series DTS 1060, Malvern Instruments S.A, Worcestershire, UK). Measurements were performed in triplicates. Furthermore, following negative staining by uranyl acetate, the morphology of NLCs and NTZ-loaded NLCs were investigated using transmission electron microscope (TEM), model JEM-100CX (JEOL, Tokyo, Japan) [22].

*IV*.*2*.*2*. *Entrapment efficiency (EE)*. Entrapment efficiency (EE) of NTZ within NLCs was evaluated at zero time and after 30 days with modified centrifugal ultrafiltration method using Centrisart-I tubes (MWCO 300 kDa, Sartorius AG, Goettingen, Germany. Briefly, the formulations (2 ml) were poured in the outer tube of Centrisart set. The set was then centrifuged using a cooling centrifuge (Sigma 3-30KS, Sigma Laborzentrifugen GmbH, Osterode, Germany) at 5000 rpm at 4 °C for 10 min. The supernatant was existing in the inner tube of Centrisart set. The NTZ amount in the supernatant was analyzed spectrophotometrically at 340 nm using Cary 60 UV-Vis Spectrophotometer, Agilent, (Santa Clara, CA, USA). The equation used to determine the EE was as follows [23]:

$$EE=\frac{Initial\;NTZ\;concentration-NTZ\;concentration\;in\;supernatant}{Initial\;NTZ\;concentration}$$

*IV*.*2*.*3*. *In vitro drug release and release kinetics*. In vitro NTZ release was carried out using dialysis technique. Appropriate volumes of NTZ-loaded NLCs equivalent to 2 mg NTZ were positioned within dialysis bags (Visking, MWCO 12,000–14,000; SERVA, Heidelberg, Germany). The bags were immersed in glass vials encompassing 10 ml of PBS with 1% sodium lauryl sulphate to ensure sink conditions, with pH adjusted to 7.4 placed in a shaking water bath (Memmert, GmbH+Co KG, Schwalbac Germany) at 37 °C, 100 rpm. Two ml of the release media were withdrawn at predetermined periods and immediately was compensated with equal volume of fresh PBS at the same temperature. The amount of NTZ was determined spectrophotometrically at 340 nm. Dialysis of free NTZ was likewise quantified under the same release conditions. To identify the release mechanism, data from release experiments were fitted to different release kinetic models (zero-order, first-order, Hixon Crowel, Higuchi, and Korsmeyer-Peppas). Experiments were done in triplicates [23].

### V. Mice infection and experimental design (Fig 1)

**Fig 1:**
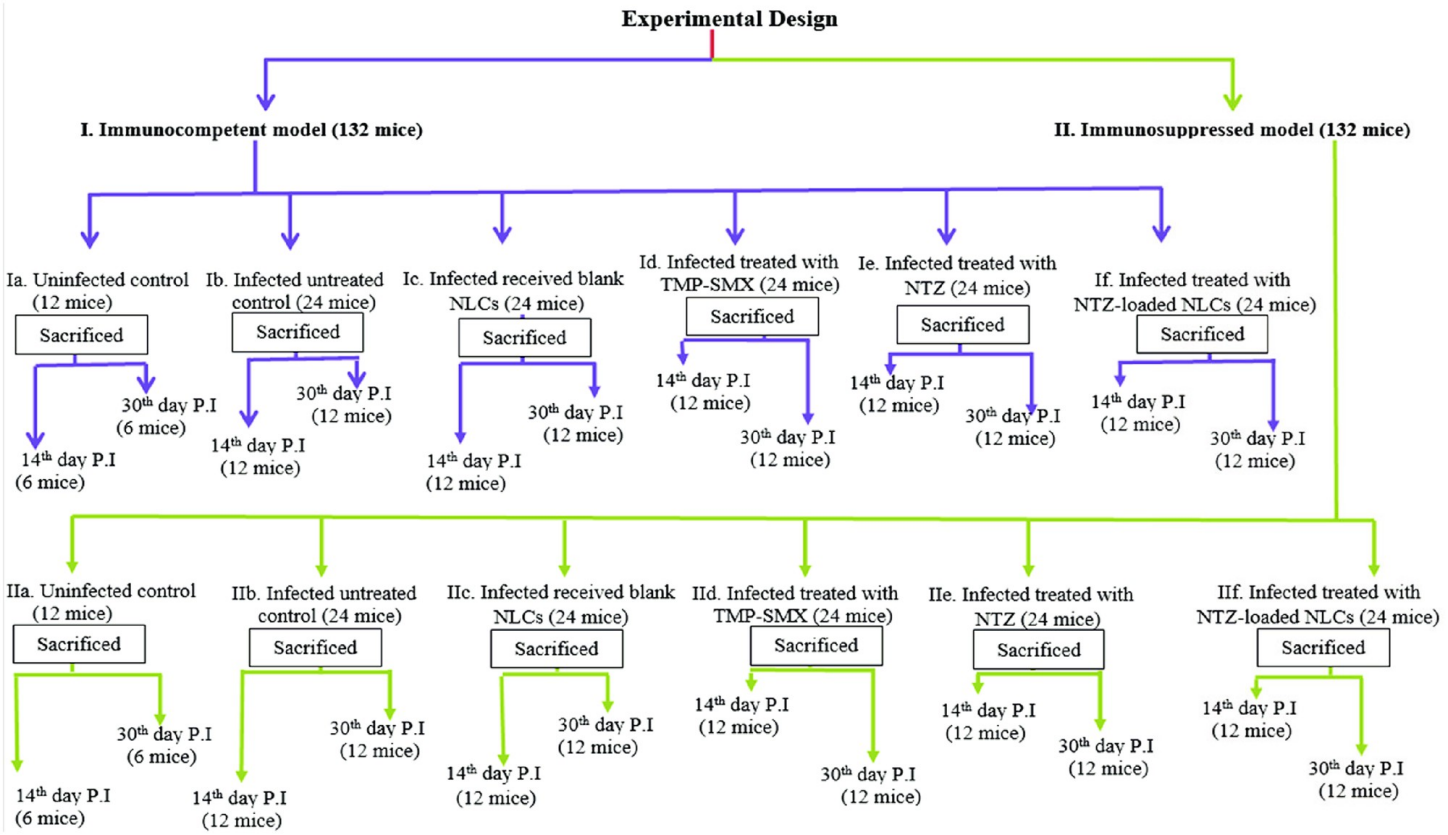
The study experimental design.

A number of 264 male Swiss Albino mice, weighing 20–25 grams, were purchased from the animal house of the Medical Parasitology Department, Faculty of Medicine, Alexandria University. Mice were housed in plastic cages under standard conditions (25–27 °C and light/dark cycles in 12-hour intervals with free access to food and drinking water).

Mice were divided into two main groups (132 mice each) as follows:

- **Group I: Immunocompetent murine model (132 mice):**

Except the normal uninfected controls, all mice were orally infected with 10^4^ sporulated oocysts (counted using Neubauer chamber) in 100 μl of phosphate buffer saline per mouse [9].

**Group I was further subdivided into**:

-**Subgroup Ia (12 mice):** Normal uninfected control where each mouse received 100 μl oral saline/day (the vehicle used for suspension of drugs) via gavage needle [9].
- **Subgroup Ib (24 mice):** *Cyclospora* infected untreated control where each mouse received 100 μl oral saline/day [9].
- **Subgroup Ic (24 mice):** Infected mice received oral blank NLCs in a dose of 320 mg/kg/day [22].
- **Subgroup Id (24 mice):** Infected mice treated with oral TMP-SMX in a dose of 5 mg/kg TMP combined with 25 mg/kg SMX one time per day [9].
- **Subgroup Ie (24 mice):** Infected mice treated with oral NTZ in a dose of 100 mg/kg/day [24].
- **Subgroup If (24 mice):** Infected mice treated with oral NTZ-loaded NLCs in a dose of 100 mg/kg/day.
  - **Group II: Immunosuppressed murine model (132 mice):**

Cyclophosphamide was given in two intraperitoneal doses separated by one week, each of 70 mg/kg/mouse to induce a state of immunosuppression in mice. One week after the second dose of cyclophosphamide, each mouse in this group (except the normal uninfected controls) was orally infected with 10^4^ sporulated oocysts in 100 μl of phosphate buffer saline [9].

**Group II was further subdivided into**:

-**Subgroup IIa (12 mice):** Normal uninfected control where each mouse received 100 μl oral saline/day [9].
- **Subgroup IIb (24 mice):** *Cyclospora* infected untreated control where each mouse received 100 μl oral saline/day [9].
- **Subgroup IIc (24 mice):** Infected mice received oral blank NLCs in a dose of 320 mg/kg/day [22].
- **Subgroup IId (24 mice):** Infected mice treated with oral TMP-SMX in a dose of 5 mg/kg TMP combined with 25 mg/kg SMX one time per day [9].
- **Subgroup IIe (24 mice):** Infected mice treated with oral NTZ in a dose of 100 mg/kg/day [24].
- **Subgroup IIf (24 mice):** Infected mice treated with oral NTZ-loaded NLCs in a dose of 100 mg/kg/day.
  - Treatment in all treated experimental subgroups started simultaneously at the beginning of oocyst shedding on the 6^th^ day post infection (P.I) and continued for 7 successive days [9].
  - Mice of normal uninfected control were divided into two equal subgroups (6 mice /each subgroup) to be sacrificed on 14^th^ and 30^th^ days P.I for histopathological assessment.
  - Mice from all infected subgroups were divided into two equal subgroups (12 mice /each subgroup). One subgroup was sacrificed on the 14^th^ day P.I for parasitological, morphological and histopathological assessment. The remaining mice (12 mice/each subgroup) were sacrificed 30 days P.I in order to evaluate the drug resistance and recurrence of infection as well as assessing the histopathological changes.

### VI. Assessment of the drug efficacy

#### VI.1. Parasitological parameters

*VI*.*1*.*1*. *Oocysts count in stool*. Fecal sample (20 mg) was collected from each mouse of the different infected subgroups at the 6^th^, 9^th^, 12^th^, 14^th^ and 30^th^ days P.I to monitor the oocysts shedding [25]. Sample from each mouse was then smeared on a glass slide and then stained by modified Ziehl Neelsen stain. The number of oocysts was counted in ten high power microscopic fields per slide by three different experienced examiners. The mean count was calculated per mouse in every subgroup. Stool samples were also stained by safranin to confirm the presence of oocysts [9,26,27].

*VI*.*1*.*2*. *Percent reduction (%R) of the parasite burden*. The % R of parasite count was estimated in each treated subgroup by using the following equation [9]:

$$\%\;R=\frac{Mean\;oocyst\;count\;in\;infected\;untreated\;control\;subgroup–Mean\;oocyst\;count\;in\;treated\;subgroup}{Mean\;oocyst\;count\;in\;infected\;untreated\;control\;subgroup}\times 100$$

#### VI.2. Ultrastructure parameter

Confirmed positive cases from all infected subgroups were collected in glutaraldehyde. Samples were further processed for scanning electron microscope (SEM) examination in order to demonstrate better morphological and structural details of the parasite [4,9,28].

#### VI.3. Histopathological parameter

Mice of the different studied subgroups were sacrificed at the 14^th^ and 30^th^ days P.I and part of small intestinal jejunum was excised and fixed in 10% neutral formalin. Serial sectioning was performed, and tissues were processed in different degrees of alcohol. They were then cleared in xylol and embedded in paraffin to form a block. On glass slides, five microns thick sections were cut by manual microtome and then stained by hematoxylin and eosin (H&E). Slides were examined to assess histopathologic alterations. Multiple photos were taken using microscope adopted camera. Villous length was measured in micro-meter (μm) by image J software in at least 10 photos from each mouse at x100 power from tip to base. Inflammation was also assessed as mild, moderate or severe in different subgroups [29]. The inflammatory cells were counted per x400 power fields using image J software. At least 6 x400 power fields in each mouse were assessed (from areas of inflammation seen in low power), then the mean value was calculated for each mouse. They were then scored as mild if up to 10 cells were counted/HPF, moderate if 11-40/HPF, severe if > 40/HPF. All histopathologic specimens used in this study were randomized, coded and assessed blindly [30].

### VII. Statistical analysis

Data were fed to the computer and analyzed using IBM SPSS software package version 20.0. (Armonk, NY: IBM Corp). For continuous data, they were tested for normality by the Shapiro-Wilk test to verify the normality of distribution of variables. Quantitative data were expressed as range (minimum and maximum), mean and standard deviation. ANOVA was used for comparing the different studied groups and followed by Post Hoc test (Tukey) for pairwise comparison while Student t-test was used to compare two main groups (immunocompetent and immunosuppressed). Significance of the obtained results was judged at the 5% level [31].

## Results

### I. Characterization of blank NLCs and NTZ-loaded NLCs

#### I.1. Colloidal properties and morphology of blank NLCs and NTZ-loaded NLCs

**Fig 2:**
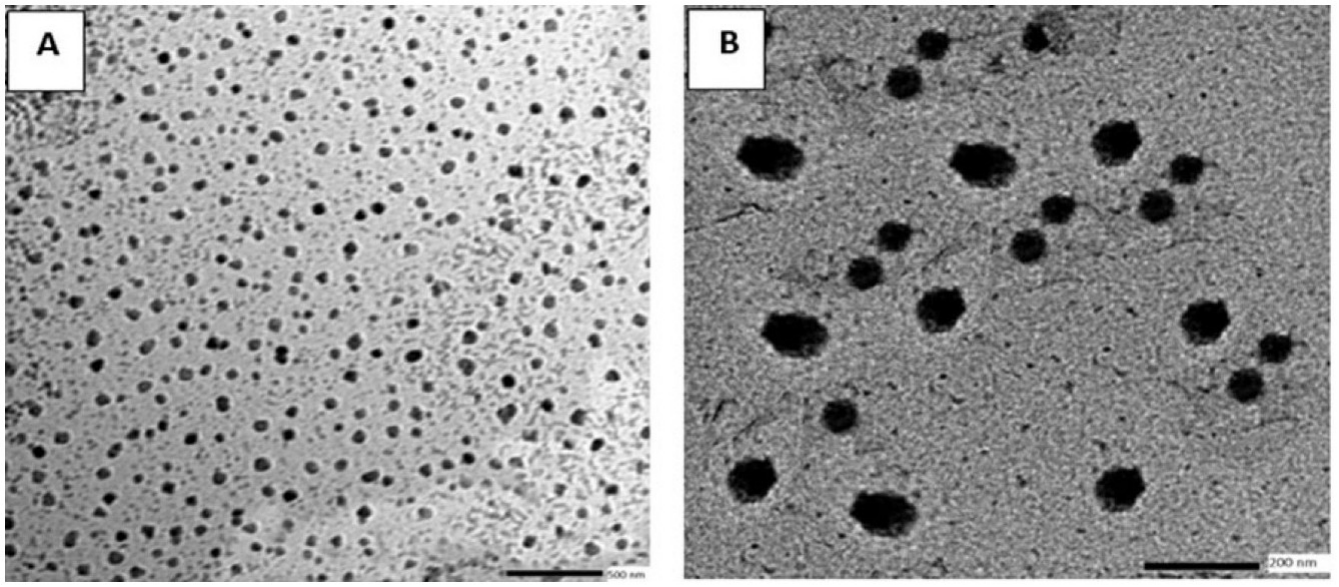
TEM micrographs of the prepared nano-formulations showing spherical shape with discrete separation. (A) Blank NLCs (scale bar = 500nm). (B) NTZ loaded NLCs (scale bar = 200nm).

The particle sizes of blank NLCs and NTZ-loaded NLCs formulations were 154 ±46 and 185 ± 67 nm respectively. PDI values were 0.25±0.02 and 0.24±0.03 for blank and NTZ-loaded NLCs respectively. Worth mentioning, the particle size and PDI of both blank and NTZ-loaded NLCs were maintained for at least 30 days at 4°C, where there was no substantial particle size increase. The zeta potential of blank NLCs was observed to be -39 mV while the zeta potential of NTZ-loaded NLCs was -36 mV. The zeta potential of both blank and NTZ-loaded NLCs didn’t change for at least 30 days. The TEM micrographs of blank NLCs showed discrete separated rounded particles. Similarly, NTZ-loaded NLCs TEM micrograph displayed almost spherical particles with neither aggregation nor fusion between particles was observed. There was no evidence of drug crystals detected as well (Fig 2).

#### I.2. Entrapment efficiency (EE)

The entrapment efficiency of NTZ within NLCs was 87.5±1% using UV spectroscopy at 340 nm with r^2^ value of 0.998. NTZ was maintained within NLCs for at least 30 days (87.5±0.9%).

#### I.3. In vitro drug release and release kinetics

**Fig 3:**
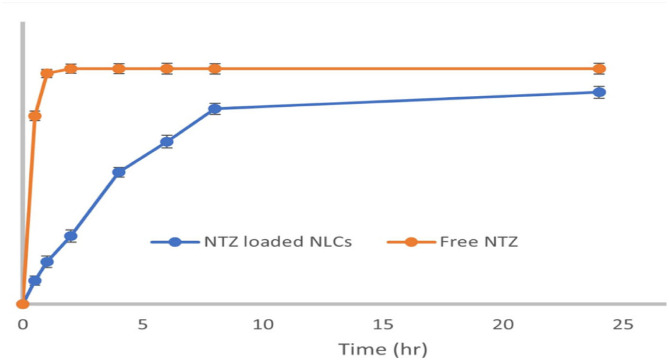
In vitro NTZ release profile from NTZ-loaded NLCs at 37 °C.

NTZ-loaded NLCs formulation revealed an initial burst release as shown in Fig 3. This may be ascribed to the leaching of NTZ from the outer layer of NLCs followed by diffusion of the drug from NLCs matrix. Up to 2 h, 29% ± 1.4 release has been observed, then NTZ release steadily increased. Up to 90%± 2.1 cumulative NTZ release was observed after 24 hr compared with almost 100% NTZ diffusion within 1 hr in case of free NTZ.

The release kinetics modeling of NTZ release best fitted first-order release kinetics with r^2^ value of 0.9932 which is greater than r^2^ values of other models; Hixson Crowel (r^2^ = 0.9897), Korsmeyer-Peppas (r^2^ = 0.9306), Higuchi (r^2^ = 0.9073) and zero order (r^2^ = 0.7864).

### II. Assessment of the drug efficacy

#### II.1. Parasitological parameters: Oocysts burden and parasite percent reduction (%R)

There was a significant increase in the oocyst shedding in infected untreated control through the whole study. Along all experimental periods, the mean oocysts count of infected untreated immunosuppressed mice was significantly higher than their corresponding immunocompetent mice till reaching 3.23 and 1.95 respectively at the end of the study.

**Fig 4:**
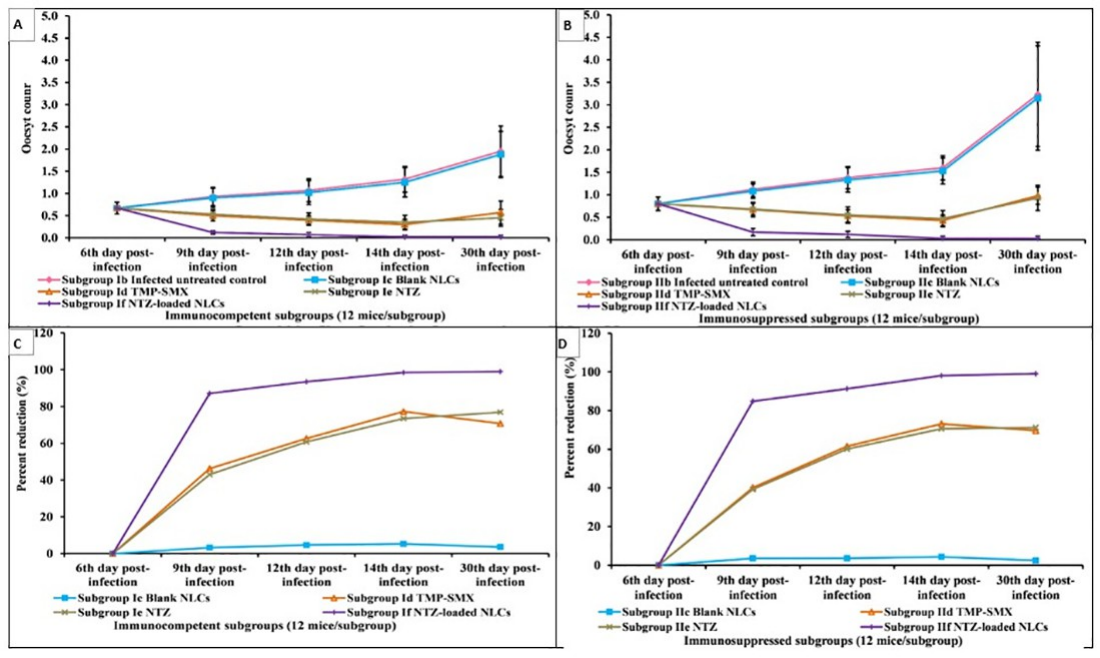
Oocysts count and parasite percent reduction in stool samples among different infected immunocompetent (A and C) and immunosuppressed (B and D) subgroups.

**Fig 5:**
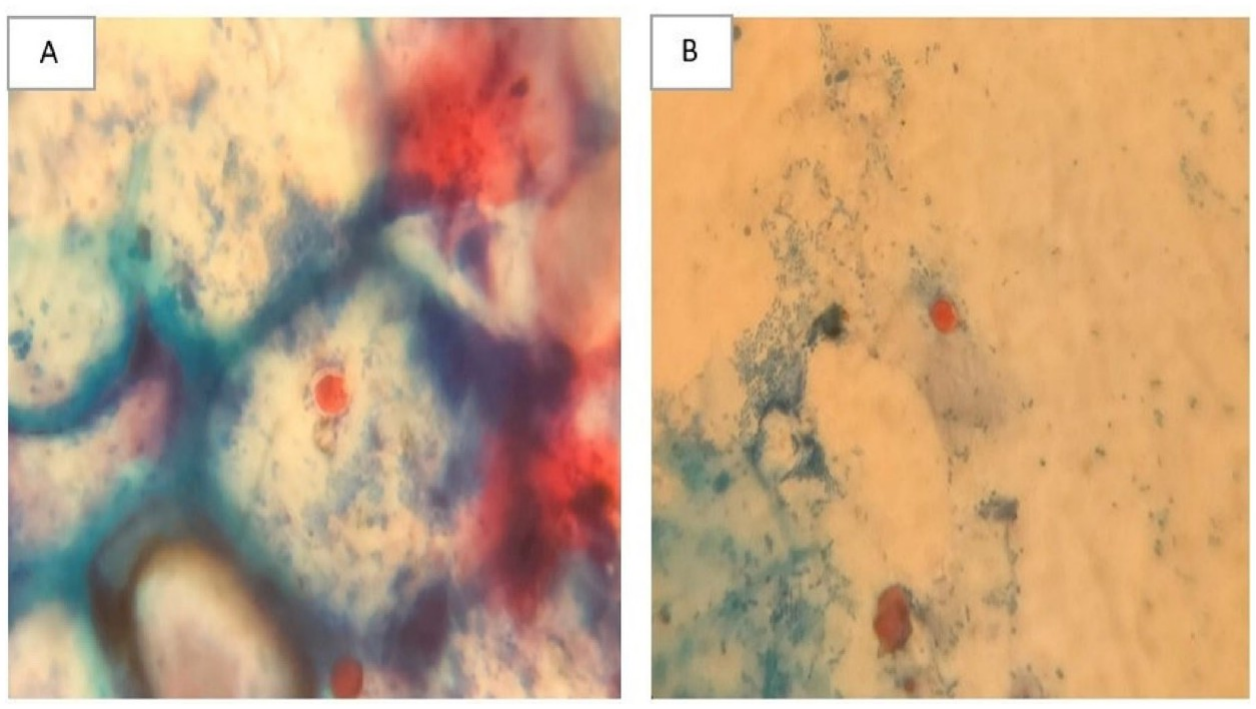
(A) and (B) Modified Ziehl Neelsen-stained stool samples revealing *Cyclospora* oocysts (X 400).

**Fig 6:**
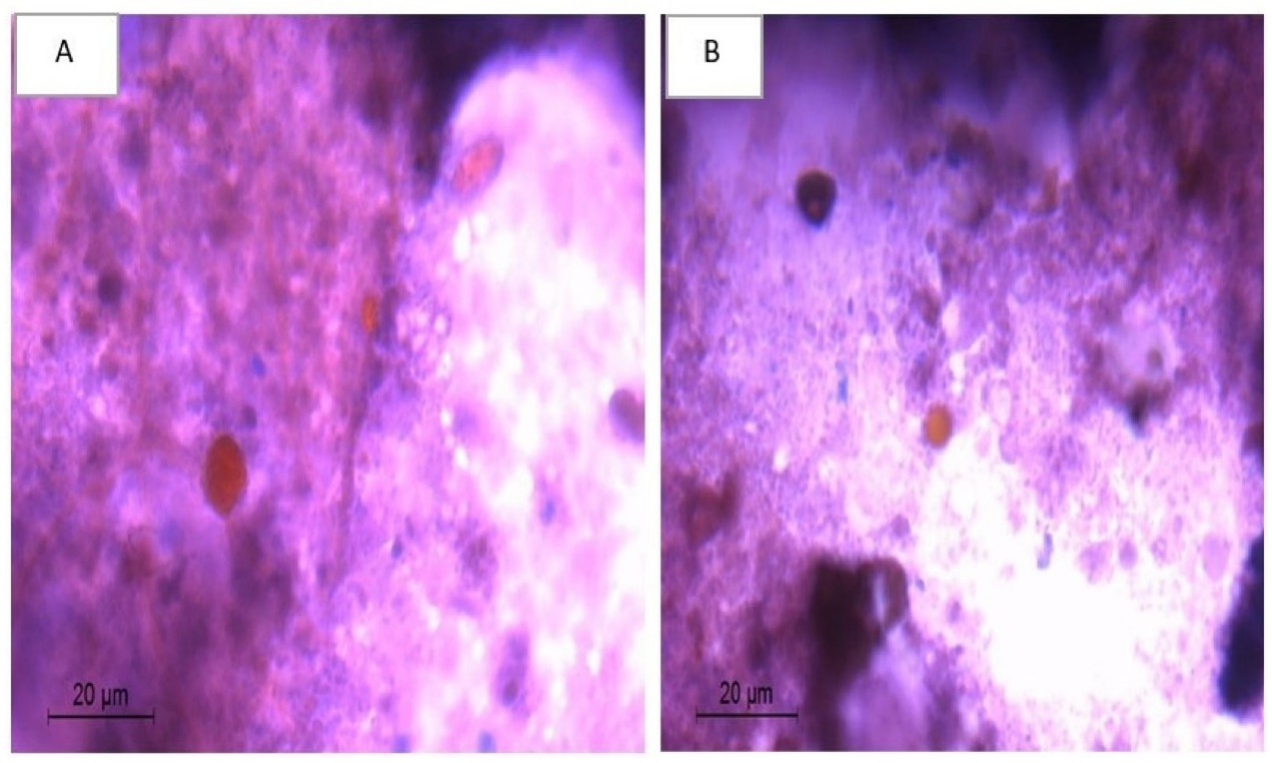
(A) and (B) Safranin-stained stool samples revealing *Cyclospora* oocysts (X 400).

Up to the 14^th^ day P.I, there was a gradual decrease in the parasite load in all infected treated subgroups (TMP-SMX, NTZ and NTZ-loaded NLCs) compared to their corresponding infected untreated control. On the 30^th^ day P.I, NTZ-loaded NLCs succeeded in avoiding parasite recurrence which was obviously noticed in all other treated subgroups. Through all study periods, NTZ-loaded NLCs treated mice showed the highest significant parasite percent reduction (i.e. lowest mean oocysts count) which exceeded 98% with no significant difference between both immunocompetent and immunosuppressed mice on both the 14^th^ and 30^th^ days P.I. (Tables 1–3 and Figs 4–6).

**Table 1:** Oocyst count and parasite percent reduction among infected immunocompetent subgroups at different examination times.

	Subgroup Ib (Infected untreated control) (n = 12)	Subgroup Ic (Blank NLCs) (n = 12)	Subgroup Id (TMP-SMX) (n = 12)	Subgroup Ie (NTZ) (n = 12)	Subgroup If (NTZ-loaded NLCs) (n = 12)
**6**^**th**^ **day P.I**					
Min.	0.50	0.50	0.50	0.50	0.50
Max.	0.80	0.80	0.80	0.80	0.80
Mean	0.67	0.67	0.67	0.67	0.67
±SD.	0.13	0.13	0.13	0.13	0.13
**F (p)**	**0.00 (1.000)**				
**% of Reduction**		**0%**	**0%**	**0%**	**0%**
**9**^**th**^ **day P.I**					
Min.	0.60	0.50	0.40	0.40	0.10
Max.	1.20	1.10	0.70	0.70	0.20
Mean	0.93	0.90	0.50^#^^@^	0.53^#^^@^	0.12^#^^@^^♦^^♣^
±SD.	0.21	0.22	0.12	0.10	0.04
**F (p)**	58.349* (<0.001*)				
**p**_**1**_		0.983	<0.001*	<0.001*	<0.001*
**p**_**2**_			<0.001*	<0.001*	<0.001*
**Sig. bet. grps.**			p_3_ = 0.983, p_4_<0.001*, p_5_<0.001*		
**% Reduction**		**3.23%**	**46.24%**	**43.01%**	**87.10%**
**12**^**th**^ **day P.I**					
Min.	0.80	0.70	0.30	0.30	0
Max.	1.40	1.30	0.60	0.70	0.10
Mean	1.07	1.02	0.40^#^^@^	0.42^#^^@^	0.07^#^^@^^♦^^♣^
±SD.	0.26	0.27	0.10	0.14	0.05
**F (p)**	65.524* (<0.001*)				
**p**_**1**_		0.964	<0.001*	<0.001*	<0.001*
**p**_**2**_			<0.001*	<0.001*	<0.001*
**Sig. bet. grps.**			p_3_ = 0.999, p_4_<0.001*, p_5_<0.001*		
**% Reduction**		**4.67%**	**62.62%**	**60.75%**	**93.46%**
**14**^**th**^ **day P.I**					
Min.	0.90	0.80	0.20	0.20	0
Max.	1.70	1.70	0.50	0.60	0.10
Mean	1.32	1.25	0.30^#^^@^	0.35^#^^@^	0.02^#^^@^^♦^^♣^
±SD.	0.29	0.33	0.12	0.16	0.04
**F (p)**	92.357* (<0.001*)				
**p**_**1**_		0.941	<0.001*	<0.001*	<0.001*
**p**_**2**_			<0.001*	<0.001*	<0.001*
**Sig. bet. grps.**			p_3_ = 0.979, p_4_ = 0.017*, p_5_ = 0.003*		
**% Reduction**		**5.30%**	**77.27%**	**73.48%**	**98.48%**
**30th day P.I**					
Min.	1.0	1.10	0.20	0.20	0
Max.	2.60	2.40	0.90	0.80	0.10
Mean	1.95	1.88	0.57^#^^@^	0.45^#^^@^	0.02^#^^@^^♦^^♣^
±SD.	0.57	0.52	0.26	0.19	0.04
**F (p)**	67.385* (<0.001*)				
**p**_**1**_		0.992	<0.001*	<0.001*	<0.001*
**p**_**2**_			<0.001*	<0.001*	<0.001*
**Sig. bet. grps.**			p_3_ = 0.940, p_4_ = 0.006*, p_5_ = 0.048*		
**% Reduction**		**3.59%**	**70.77%**	**76.92%**	**98.97%**

SD: **Standard deviation**

**% Reduction:** relative to infected untreated control of each study period

**F**: **F for ANOVA test**. Pairwise comparison between each 2 groups was done using **Post Hoc Test (Tukey)**

p: p value for comparing between the studied groups

p_1_: p value for comparing between immunocompetent **infected untreated control** and each other group

p_2_: p value for comparing between immunocompetent **Blank NLCs** and each other group

p_3_: p value for comparing between immunocompetent **TMP-SMX** and **NTZ**

p_4_: p value for comparing between immunocompetent **TMP-SMX** and **NTZ-loaded NLCs**

p_5_: p value for comparing between immunocompetent **NTZ** and **NTZ-loaded NLCs**

*: Statistically significant at p ≤ 0.05

^#^: Significant with infected untreated control

^@^: Significant with blank NLCs

^♦^: Significant with TMP-SMX

^♣^: Significant with NTZ

**Table 2:** Oocyst count and parasite percent reduction among infected immunosuppressed subgroups at different examination times.

	Subgroup IIb (Infected untreated control) (n = 12)	Subgroup IIc (Blank NLCs) (n = 12)	Subgroup IId (TMP-SMX) (n = 12)	Subgroup IIe (NTZ) (n = 12)	Subgroup IIf (NTZ-loaded NLCs) (n = 12)
**6**^**th**^ **day P.I**					
Min.	0.70	0.70	0.70	0.70	0.70
Max.	1	1	1	1	1
Mean	0.80	0.80	0.80	0.80	0.80
±SD.	0.15	0.15	0.15	0.15	0.15
**F (p)**	**1.000 (0.998)**				
**% Reduction**		**0%**	**0%**	**0%**	**0%**
**9**^**th**^ **day P.I**					
Min.	0.90	0.80	0.50	0.60	0.10
Max.	1.40	1.30	0.90	0.90	0.30
Mean	1.12	1.08	0.67^#^^@^	0.68^#^^@^	0.17^#^^@^^♦^^♣^
±SD.	0.16	0.16	0.16	0.13	0.08
**F (p)**	89.279* (<0.001*)				
**p**_**1**_		0.978	<0.001*	<0.001*	<0.001*
**p**_**2**_			<0.001*	<0.001*	<0.001*
**Sig. bet. grps.**			p_3_ = 0.998, p_4_<0.001*, p_5_<0.001*		
**% Reduction**		**3.57%**	**40.18%**	**39.29%**	**84.82%**
**12**^**th**^ **day P.I**					
Min.	1	0.90	0.40	0.30	0
Max.	1.70	1.60	0.80	0.80	0.20
Mean	1.38	1.33	0.53^#^^@^	0.55^#^^@^	0.12^#^^@^^♦^^♣^
±SD.	0.25	0.27	0.14	0.18	0.07
**F (p)**	95.406* (<0.001*)				
**p**_**1**_		0.971	<0.001*	<0.001*	<0.001*
**p**_**2**_			<0.001*	<0.001*	<0.001*
**Sig. bet. grps.**			p_3_ = 1.000, p_4_<0.001*, p_5_<0.001*		
**% Reduction**		**3.62%**	**61.59%**	**60.14%**	**91.3%**
**14**^**th**^ **day P.I**					
Min.	1.20	1.10	0.30	0.30	0
Max.	2	1.90	0.60	0.80	0.10
Mean	1.60	1.53	0.43^#^^@^	0.47^#^^@^	0.03^#^^@^^♦^^♣^
±SD.	0.27	0.29	0.12	0.18	0.05
**F (p)**	148.961* (<0.001*)				
**p**_**1**_		0.926	<0.001*	<0.001*	<0.001*
**p**_**2**_			<0.001*	<0.001*	<0.001*
**Sig. bet. grps.**			p_3_ = 0.994, p_4_<0.001*, p_5_<0.001*		
**% Reduction**		**4.38%**	**73.13%**	**70.63%**	**98.13%**
**30th day P.I**					
Min.	1.40	1.30	0.60	0.40	0
Max.	4.90	4.80	1.10	1.20	0.10
Mean	3.23	3.15	0.98^#^^@^	0.93^#^^@^	0.03^#^^@^^♦^^♣^
±SD.	1.16	1.16	0.19	0.28	0.05
**F (p)**	44.313* (<0.001*)				
**p**_**1**_		0.999	<0.001*	<0.001*	<0.001*
**p**_**2**_			<0.001*	<0.001*	<0.001*
**Sig. bet. grps.**			p_3_ = 1.000, p_4_ = 0.024*, p_5_ = 0.037*		
**% Reduction**		**2.48%**	**69.66%**	**71.21%**	**99.07%**

SD: **Standard deviation**

**% Reduction:** relative to infected untreated control of each study period

**F**: **F for ANOVA test**. Pairwise comparison between each 2 groups was done using **Post Hoc Test (Tukey)**

p: p value for comparing between the studied groups

p_1_: p value for comparing between immunosuppressed **infected untreated control** and each other group

p_2_: p value for comparing between immunosuppressed **Blank NLCs** and each other group

p_3_: p value for comparing between immunosuppressed **TMP-SMX** and **NTZ**

p_4_: p value for comparing between immunosuppressed **TMP-SMX** and **NTZ-loaded NLCs**

p_5_: p value for comparing between immunosuppressed **NTZ** and **NTZ-loaded NLCs**

*: Statistically significant at p ≤ 0.05

^#^: Significant with infected untreated control

^@^: Significant with blank NLCs

^♦^: Significant with TMP-SMX

^♣^: Significant with NTZ

**Table 3:** Comparison between the immunocompetent and immunosuppressed groups regarding oocyst count at different examination times.

	Infected untreated control (n = 12)		Blank NLCs (n = 12)		TMP-SMX (n = 12)		NTZ (n = 12)		NTZ-loaded NLCs (n = 12)	
	Group I (IC)	Group II (IS)	Group I (IC)	Group II (IS)	Group I (IC)	Group II (IS)	Group I (IC)	Group II (IS)	Group I (IC)	Group II (IS)
**6**^**th**^ **day P.I**										
Min.	0.50	0.70	0.50	0.70	0.50	0.70	0.50	0.70	0.50	0.70
Max.	0.80	1	0.80	1	0.80	1	0.80	1	0.80	1
Mean	0.67	0.80	0.67	0.80	0.67	0.80	0.67	0.80	0.67	0.80
± SD.	0.13	0.15	0.13	0.15	0.13	0.15	0.13	0.15	0.13	0.15
**t (p)**	2.345* (0.028*)		2.345* (0.028*)		2.345* (0.028*)		2.345* (0.028*)		2.345* (0.028*)	
**9**^**th**^ **day P.I**										
Min.	0.60	0.90	0.50	0.80	0.40	0.50	0.40	0.60	0.10	0.10
Max.	1.20	1.40	1.10	1.30	0.70	0.90	0.70	0.90	0.20	0.30
Mean	0.93	1.12	0.90	1.08	0.50	0.67	0.53	0.68	0.12	0.17
± SD.	0.21	0.16	0.22	0.16	0.12	0.16	0.10	0.13	0.04	0.08
**t (p)**	2.411* (0.025*)		2.331* (0.029*)		2.932* (0.008*)		3.238* (0.004*)		1.990 (0.064)	
**12**^**th**^ **day P.I**										
Min.	0.80	1	0.70	0.90	0.30	0.40	0.30	0.30	0	0
Max.	1.40	1.70	1.30	1.60	0.60	0.80	0.70	0.80	0.10	0.20
Mean	1.07	1.38	1.02	1.33	0.40	0.53	0.42	0.55	0.07	0.12
± SD.	0.26	0.25	0.27	0.27	0.10	0.14	0.14	0.18	0.05	0.07
**t (p)**	3.028* (0.006*)		2.910* (0.008*)		2.602* (0.016*)		2.035 (0.054)		1.990 (0.059)	
**14**^**th**^ **day P.I**										
Min.	0.90	1.20	0.80	1.10	0.20	0.30	0.20	0.30	0	0
Max.	1.70	2	1.70	1.90	0.50	0.60	0.60	0.80	0.10	0.10
Mean	1.32	1.60	1.25	1.53	0.30	0.43	0.35	0.47	0.02	0.03
± SD.	0.29	0.27	0.33	0.29	0.12	0.12	0.16	0.18	0.04	0.05
**t (p)**	2.499* (0.020*)		2.248* (0.035*)		2.766* (0.011*)		1.707 (0.102)		0.920 (0.368)	
**30**^**th**^ **day P.I**										
Min.	1.0	1.40	1.10	1.30	0.20	0.60	0.20	0.40	0	0
Max.	2.60	4.90	2.40	4.80	0.90	1.10	0.80	1.20	0.10	0.10
Mean	1.95	3.23	1.88	3.15	0.57	0.98	0.45	0.93	0.02	0.03
± SD.	0.57	1.16	0.52	1.16	0.26	0.19	0.19	0.28	0.04	0.05
**t (p)**	3.428* (0.003*)		3.453* (0.003*)		4.517* (<0.001*)		4.954* (<0.001*)		0.920 (0.368)	

SD: **Standard deviation**

t: Student t-test

IC: immunocompetent

IS: immunosuppressed

p: p value for comparing between **group I (immunocompetent)** and **group II (immunosuppressed)**

*: Statistically significant at p ≤ 0.05

#### II.2. Ultrastructure parameter by SEM

**Fig 7:**
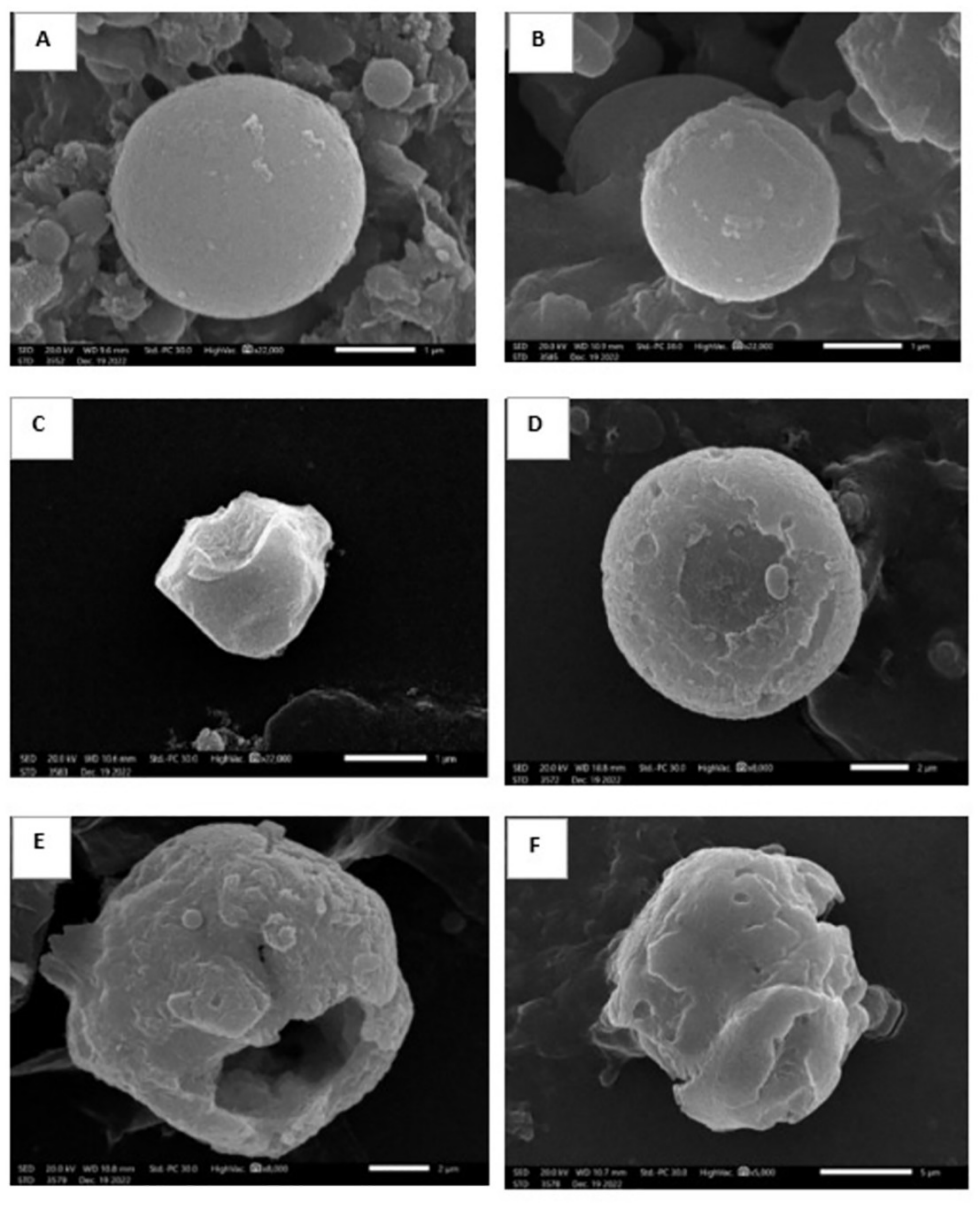
SEM of *Cyclospora* oocysts collected from different studied subgroups of the immunocompetent murine model. (A) Oocyst from subgroup I b (infected untreated control), revealing spherical shape with smooth regular surface (X 22,000). (B) Oocyst from subgroup I c (receiving blank NLCs), showing spherical smooth surface organism (X 22,000). (C) Oocyst from subgroup I d (treated with TMP-SMX), showing cauliflower-like shrunken parasite losing its smooth surface with noticeable furrows and ridges (X 22,000). (D) Oocyst from subgroup I e (treated with NTZ), showing a swollen distorted parasite losing the smooth surface with apparent surface erosion and ulceration (X 8,000). (E) and (F) Oocysts from subgroup I f (treated with NTZ-loaded NLCs), showing mutilated rupturing parasite losing its spherical shape and smooth surface with multiple blebs, clefts, lacerations and profound cavitations on the parasite surface (X 8,000 and 5,000).

**Fig 8:**
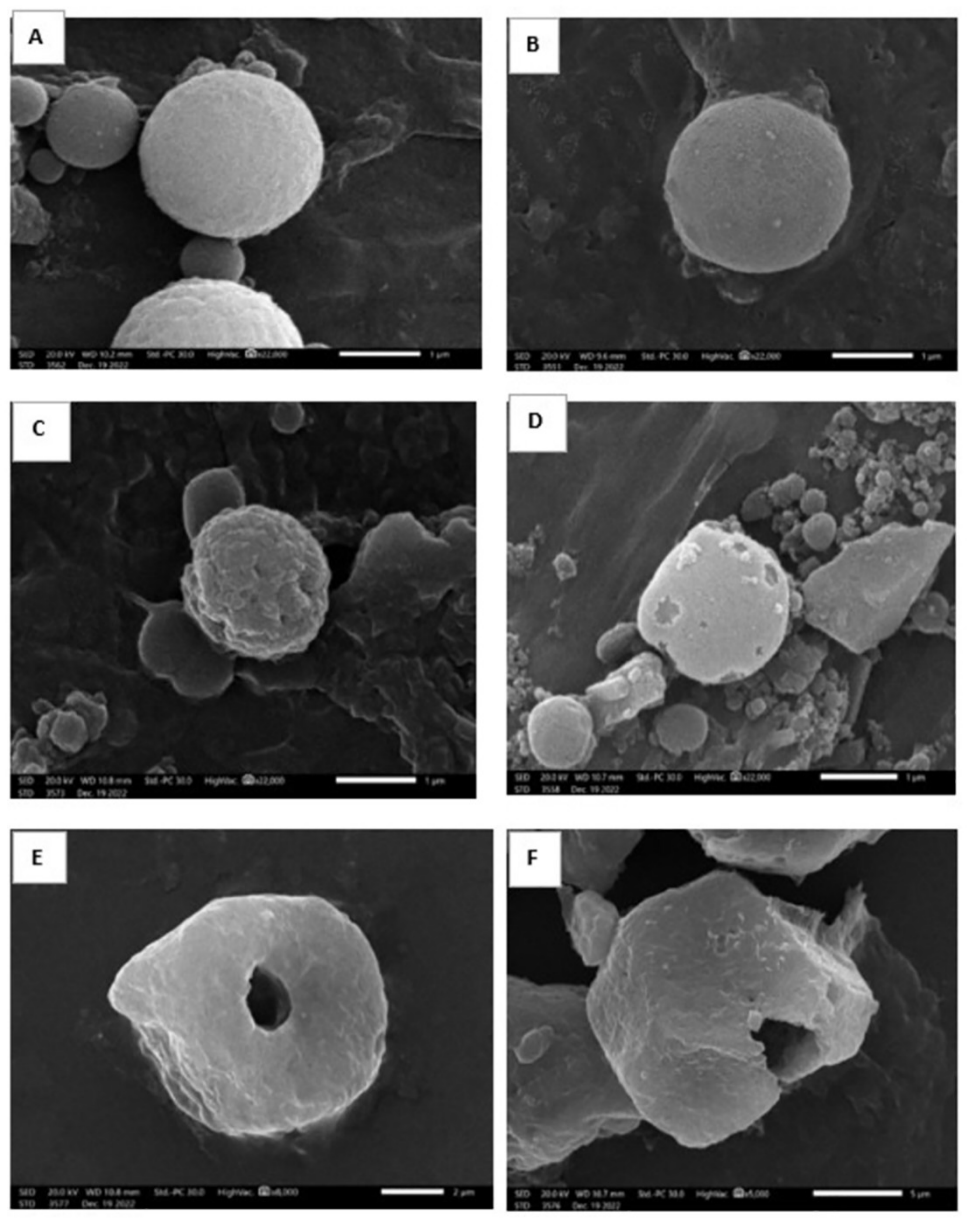
SEM of *Cyclospora* oocysts collected from different studied subgroups of the immunosuppressed murine model. (A) Oocyst from subgroup II b (infected untreated control), showing a spherical-shaped organism with smooth regular surface (X 22,000). (B) Oocyst from subgroup II c (receiving blank NLCs), demonstrating spherical smooth surface parasite (X 22,000). (C) Oocyst from subgroup II d (treated with TMP-SMX), revealing dimpled irregular outer surface (X 22,000). (D) Oocyst from subgroup II e (treated with NTZ), showing a disorganized organism with noticeable multiple deep perforations (X 22,000). (E) and (F) Oocysts from subgroup II f (treated with NTZ-loaded NLCs), showing a disfigured rupturing parasite losing its spherical shape and smooth surface with deep cavitations (X 8,000 and 5,000).

SEM was used to study the ultrastructure of oocysts. Oocysts of infected untreated control in both immunocompetent and immunosuppressed mice were spherical with an outer regular smooth surface. Nevertheless, oocysts collected from all treated subgroups (TMP-SMX, NTZ and NTZ-loaded NLCs) appeared distorted with loss of smooth surface, furrows, ridges, erosions, ulcerations, blebs, clefts, lacerations, perforations and/or cavitations. The changes were more apparent in mice treated with NTZ-loaded NLCs where the oocysts revealed remarkable mutilation and rupturing. All morphological changes were more apparent in immunocompetent group (Figs 7 and 8).

#### II.3. Histopathological parameter

**Fig 9:**
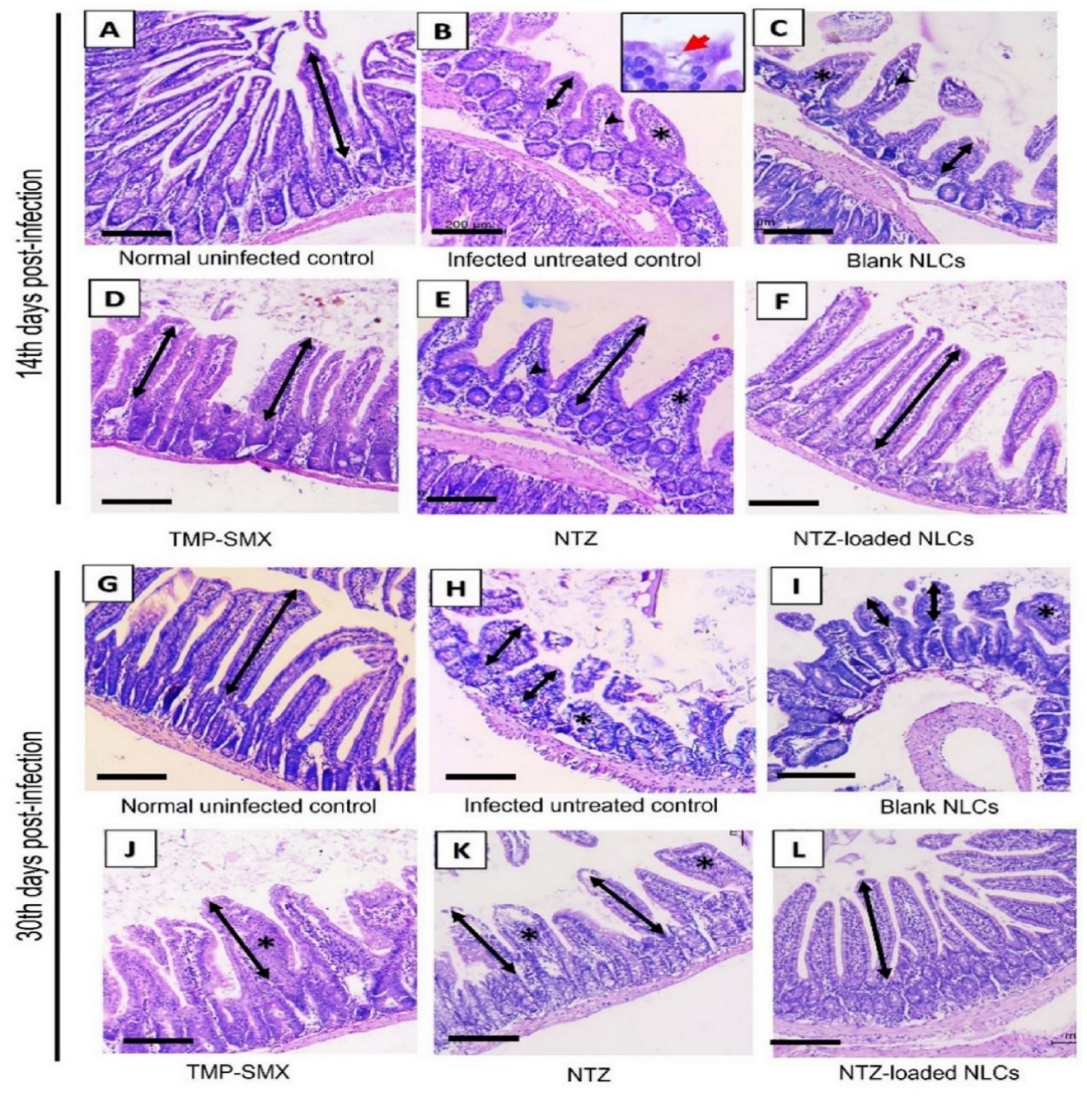
Intestinal histopathologic changes of immunocompetent group on the 14^th^ and 30^th^ days P.I. (H&E, x100, scale bar = 200 μm). 14^th^ day P.I: (A) Normal intestinal histology is seen in normal uninfected control group. (B) Infected untreated subgroup showing short (bi-headed arrow) broad edematous villi with dilated capillaries (arrow head) as well as severe inflammation (*). Inset (x1000) shows the organism within enterocyte (red arrow). (C) The subgroup receiving blank NLCs demonstrating similar histopathological findings as in infected untreated control. (D) TMP-SMX treated subgroup reveals increased villous length (bi-headed arrow) with improved inflammation. (E) In NTZ treated subgroup, increased villous length (bi-headed arrow) is observed but moderate inflammation is still present (*) as well as congested capillaries (arrow head). (F) NTZ-loaded NLCs subgroup presents long slender villi (bi-headed arrow) with no significant inflammation. 30^th^ day post infection: (G) Normal histology is apparent in normal uninfected subgroup. (H) and (I) The pathologic changes are exaggerated in infected untreated control and blank NLCs subgroups respectively. (J) In TMP-SMX subgroup, moderate inflammation is noticed back again (*). (K) In NTZ treated subgroup, moderate inflammation is seen (*). (L) NTZ-loaded NLCs subgroup shows restoration of normal villous length and lack of inflammation. *Bi-headed arrow = villous length*, * = inflammation, *arrow head = dilated capillaries*.

*II.3.1. Histopathological study in immunocompetent subgroups (Table 4 and* Fig 9*)*. Normal uninfected immunocompetent mice revealed preserved villous border. The villi were long and slender, lined by long enterocytes. Similar mean villous height was observed on both the 14^th^ and 30^th^ days P.I (426.4 μm).

**Table 4:** Comparison between the different studied immunocompetent subgroups according to intestinal villi length on the 14^th^ and 30^th^ days P.I.

	Subgroup Ia (Normal uninfected control)	Subgroup Ib (Infected untreated control)	Subgroup Ic (Blank NLCs)	Subgroup Id (TMP-SMX)	Subgroup Ie (NTZ)	Subgroup If (NTZ-loaded NLCs)
**14**^**th**^ **day P.I**						
Min. (μm)	388.8	122.1	111.9	238.80	182.3	342
Max. (μm)	456.1	218.2	268.8	338.6	343.4	456
Mean (μm)	426.4	166.5^#^	191.8^#^	303.8^#^^@^^♦^	266.4^#^^@^^♦^	380.5^@^^♦^^♣^^&^
±SD (μm)	27.9	37.2	51.6	31.5	50	35.8
**F (p)**	78.669* (<0.001*)					
**p**_**1**_		<0.001*	<0.001*	<0.001*	<0.001*	0.068
**p**_**2**_			0.634	<0.001*	<0.001*	<0.001*
**p**_**3**_				<0.001*	<0.001*	<0.001*
**Sig. bet. grps.**				p_4_ = 0.212, p_5_<0.001*, p_6_<0.001*		
**30th day P.I**						
Min. (μm)	388.8	110	95.5	221.3	236.8	338.8
Max. (μm)	456.1	201.9	199.8	363	315.6	456
Mean(μm)	426.4	147.8^#^	137.2^#^	299.9^#^^@^^♦^	279.9^#^^@^^♦^	390.6^@^^♦^^♣^^&^
±SD (μm)	27.9	33.5	30.3	39	24	45.4
**F (p)**	148.414* (<0.001*)					
**p**_**1**_		<0.001*	<0.001*	<0.001*	<0.001*	0.119
**p**_**2**_			0.972	<0.001*	<0.001*	<0.001*
**p**_**3**_				<0.001*	<0.001*	<0.001*
**Sig. bet. grps.**				p_4_ = 0.704, p_5_<0.001*, p_6_<0.001*		

SD: **Standard deviation**

**F**: **F for ANOVA test**. Pairwise comparison bet. each 2 groups was done using **Post Hoc Test (Tukey)**

p: p value for comparing between the studied groups

p_1_: p value for comparing between **immunocompetent normal uninfected control** and each other group

p_2_: p value for comparing between **immunocompetent infected untreated control** and each other group

p_3_: p value for comparing between **immunocompetent blank NLCs** and each other group

p_4_: p value for comparing between **immunocompetent TMP-SMX** and **NTZ**

p_5_: p value for comparing between **immunocompetent TMP-SMX** and **NTZ-loaded NLCs**

p_6_: p value for comparing between **immunocompetent NTZ** and **NTZ-loaded NLCs**

*: Statistically significant at p ≤ 0.05

^#^: Significant with normal uninfected control

^@^: Significant with infected untreated control

^♦^: Significant with blank NLCs

^♣^: Significant with TMP-SMX

^&^: Significant with NTZ

On the 14^th^ day P.I, infected untreated immunocompetent mice showed alteration of normal intestinal architecture with atrophic short villi. They were broad and edematous. Their mean length was 166.5 μm. They revealed denuded surface epithelium in some foci. The crypts were hyperplastic with increased mitotic activity. Moderate to severe inflammation was also seen in villi and lamina propria with dilated villous capillaries. After 30 days, the changes were exaggerated with overstated villous atrophy (147.8 μm).

The infected mice which received blank NLCs demonstrated same the pathological changes. The villi were still atrophic with evident severe inflammation in both 14^th^ and 30^th^ days samples.

The infected subgroup which received TMP-SMX showed improvement of intestinal histology. The mean villous length increased up to 303.8 μm with mild inflammation. After 30 days, the length slightly decreased to 299.9 μm and the villi appeared broad, edematous with moderate inflammation.

NTZ treated mice showed improvement of the mean villous length (266.4 μm) compared to infected untreated control. However, congested capillaries with moderate inflammation were still seen in the villous core. After 30 days, similar histological changes were observed.

Meanwhile, loading NTZ on NLCs augmented its therapeutic efficiency. The mean villous length significantly increased up to 380.5 and 390.6 μm on the 14^th^ and 30^th^ days respectively, showing no significant difference with the normal uninfected control. The villi were long and slender, with no signs of edema or congestion. No significant inflammatory changes were detected in day 14 as well as day 30 specimens.

**Fig 10:**
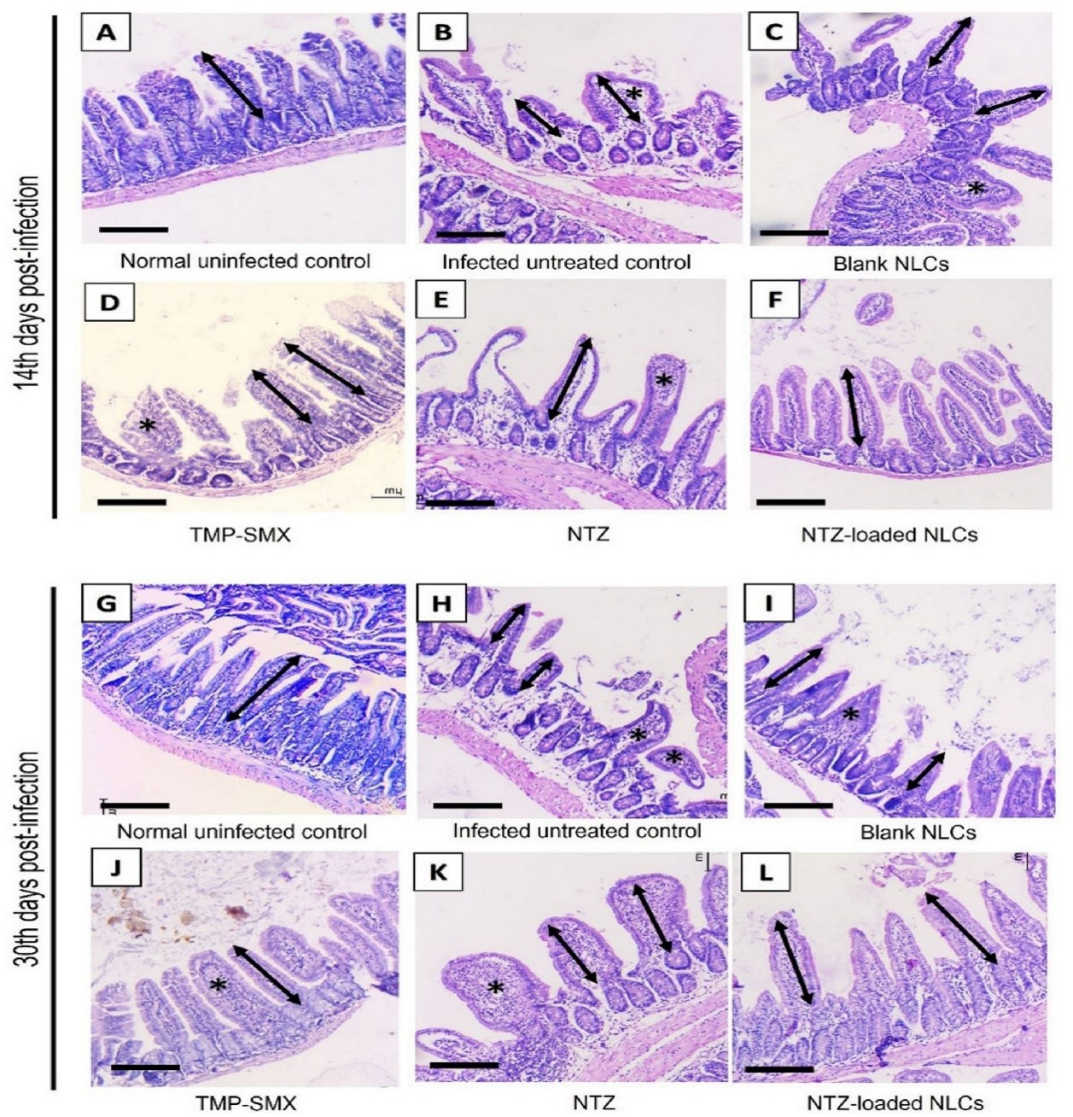
Intestinal histopathologic changes of immunosuppressed group on 14^th^ and 30^th^ days P.I. (H&E, x100, scale bar = 200 μm). In 14^th^ days post infection: (A) Normal uninfected control reveals normal intestinal architecture. (B) and (C) Both infected untreated control and blank NLCs subgroups present short (bi-headed arrow) broad edematous villi as well as inflammatory changes (*). (D) TMP-SMX treated mice shows increased villous length with moderate inflammation in some villi (*). (E) NTZ treated subgroup displays increased villous length, however moderate inflammation is still existing (*). (F) The subgroup treated with NTZ-loaded NLCs demonstrates restoration of the normal long slender villous morphology. In 30^th^ days post infection: (G) Normal histology is still seen in normal uninfected group. (H) and (I) More apparent pathologic changes of *Cyclospora* infection are noticed in infected untreated and blank NLCs subgroups respectively. (J) Residual inflammation (*) is still observed in some villi in TMP-SMX treated mice. (K) NTZ treated subgroup shows similar pathologic findings as TMP-SMX. (L) Restoration of villous length is still obviously noted in NTZ-loaded NLCs with no evident inflammation. *Bi-headed arrow = villous length*, * = inflammation.

*II*.*3*.*2*. *Histopathological study in immunosuppressed subgroups (*Table 5 *and* Fig 10*)*. Normal uninfected immunosuppressed mice demonstrated mild shortening of villi in comparison to their corresponding immunocompetent mice in both the 14^th^ and 30^th^ day samples (345.5 μm).

**Table 5:** Comparison between the different studied immunosuppressed subgroups according to the intestinal villi length on the 14^th^ and 30^th^ days P.I.

	Subgroup IIa (Normal uninfected control)	Subgroup IIb (Infected untreated control)	Subgroup IIc (Blank NLCs)	Subgroup IId (TMP-SMX)	Subgroup IIe (NTZ)	Subgroup IIf (NTZ-loaded NLCs)
**14**^**th**^ **day P.I**						
Min. (μm)	299	117.7	120	177.6	195.3	285.3
Max. (μm)	399	287.4	220.7	324.6	323	400
Mean (μm)	345.5	201.3^#^	165^#^	253.3^#^^♦^	251.3^#^^♦^	327.3^@^^♦^^♣^^&^
±SD (μm)	32.3	67.2	36.8	46	40.6	37.7
**F (p)**	21.803* (<0.001*)					
**p**_**1**_		<0.001*	<0.001*	0.004*	0.001*	0.954
**p**_**2**_			0.512	0.204	0.177	<0.001*
**p**_**3**_				0.004*	0.003*	<0.001*
**Sig. bet. grps.**						
**30th day P.I**						
Min. (μm)	299	95.5	136.9	175	185	258.3
Max. (μm)	399	198	204.7	320.3	364.5	418.5
Mean (μm)	345.5	149.8^#^	166.1^#^	247.3^#^^@^^♦^	255.4^#^^@^^♦^	335^@^^♦^^♣^^&^
±SD (μm)	32.3	29.6	26.9	57.6	71.5	54.3
**F (p)**	20.233* (<0.001*)					
**p**_**1**_		<0.001*	<0.001*	0.002*	0.004*	0.999
**p**_**2**_			0.988	0.002*	<0.001*	<0.001*
**p**_**3**_				0.026*	0.008*	<0.001*
**Sig. bet. grps.**				p_4_ = 0.999, p_5_ = 0.013*, p_6_ = 0.023*		

SD: **Standard deviation**

**F**: **F for ANOVA test**. Pairwise comparison bet. each 2 groups was done using **Post Hoc Test (Tukey)**

p: p value for comparing between the studied groups

p_1_: p value for comparing between **immunosuppressed normal uninfected control** and each other group

p_2_: p value for comparing between **immunosuppressed infected untreated control** and each other group

p_3_: p value for comparing between **immunosuppressed blank NLCs** and each other group

p_4_: p value for comparing between **immunosuppressed TMP-SMX** and **NTZ**

p_5_: p value for comparing between **immunosuppressed TMP-SMX** and **NTZ-loaded NLCs**

p_6_: p value for comparing between **immunosuppressed NTZ** and **NTZ-loaded NLCs**

*: Statistically significant at p ≤ 0.05

^#^: Significant with normal uninfected control

^@^: Significant with infected untreated control

^♦^: Significant with blank NLCs

^♣^: Significant with TMP-SMX

^&^: Significant with NTZ

Immunosuppressed infected untreated mice presented similar pathologic changes to the immunocompetent group. Nevertheless, villous shortening was more diffuse and lower inflammatory response was seen compared to their corresponding immunocompetent subgroups in both the 14^th^ and 30^th^ days specimens. Similar changes were seen in blank NLCs treated mice with no improvement seen.

Both TMP-SMX and NTZ treated immunosuppressed mice showed moderate improvement of villous length. Though, the most pronounced pathologic improvement was observed in NTZ-loaded NLCs treated mice. Compared to the normal uninfected control, NTZ-loaded NLCs is the only subgroup that showed no significant difference in villi length revealing the success in approaching the normal histology.

## Discussion

Cyclosporiasis is one of the foremost public health issues in the world with more abundance in tropical and subtropical regions [1–3,29]. The disease denotes an enormous burden on human health where symptoms range from mild to fatal depending on the age and immune status [3]. There are several therapeutic options for *C*. *cayetanensis*. Meanwhile, a well-tolerated treatment with high efficiency and low resistant rate is still a true challenge [9]. NTZ is one of the most commonly used drugs for treatment of intestinal protozoa and helminths presenting an extensive tolerance level. However, it showed failure rates of 13–29% in the treatment of cyclosporiasis and 20% in the treatment of giardiasis [3,32]. Concurrently, most of the immunocompromised patients with cryptosporidiosis did not respond well with a high rate of infection recurrence upon treatment with NTZ [33,34]. Recently, NTZ showed a partial success when used as an alternative treatment to triclabendazole failure in the management of acute fascioliasis [35]. In this respect, there is a crucial urgency for developing novel alternatives to improve the therapeutic benefits of the available drugs [9].

Nanotechnology offers a new frontier in drug delivery systems to surmount the limitations accompanied with the conventional treatment regimens [36]. Nanomaterials rely on the fact that the thickness of the intestinal mucosal layer is about 700 μm and the pore size of the absorptive diffusion channels is ranging from 20 to 200 nm [37]. Subsequently, nanoparticles with a size up to hundreds of nanometers can diffuse easily through the mucus membranes in just few minutes. Accordingly, this would enhance the permeability and bioavailability of the used drugs [38]. Nano-sized particles concurrently ease the penetration of the loaded drug through the outer surface of micro-organisms, adding a value in improving the efficiency of commercial drugs [39]. On top of that, another benefit of using nano-vehicles is the sustained-release action of the incorporated drug leading to the reduction of infection recurrence which sequentially presents a cost-effective method on the long run. Although identifying new therapeutic agents is of great priority, the development of the nanocarrier for commercially available drugs may represent a more cost-effective strategy [40]. Lipid-based nanocarriers have gained substantial reputation for their safe nature which is approved by FDA, in addition to their potential ability of increasing the solubility of lipophilic drugs. Besides, NLCs as a lipid nanocarrier have shown a promising potential application in oral drug delivery systems. Controlled drug delivery biocompatible system like NLCs could be beneficial to reduce the drug dose and its frequency of dosing and thus lowering the possible toxicity by the free drug [14–17].

The main objective of the present study was to prepare a novel formula composed of a lipid nanocarrier (NLCs) to deliver the loaded drug (NTZ) in a nanometric size in order to enhance the biological activity and tissue permeability of the drug. Immunocompetent and immunosuppressed models of murine cyclosporiasis were used to evaluate the treatment efficacy.

Previous studies have loaded NTZ within liposomes and solid lipid nanoparticles [41–43]. Herein, NTZ was successfully loaded within NLCs. Particle size increased after NTZ incorporation implying that the loaded NTZ is entangled in aliphatic chains of fatty acids and triglycerides and is entrapped within the matrix of NLCs rather than adsorbed on NLCs surface. This was confirmed by TEM images where there is no incidence of drug particles or crystals at NLCs surface. NLCs displays advanced drug loading capacity in comparison with solid lipid nanoparticles. Moreover, it is reported that the drug loading capacity would increase with increasing oleic acid amount. The presence of oleic acid within NLCs would lead to substantial crystal order disruption and formation of less ordered NLCs matrix and thus, enhancing the drug loading capacity. Also, the presence of oleic acid in the formulation could improve the formation of smaller nanoparticles with homogenous particle size distributions [44]. Lebrafil was reported to induce the formation of less viscous formulations and reduce the surface tension which would lead to formation of smaller nanoparticles [45]. Both oleic acid and Lebrafil would ensure better dispersity of NTZ within NLCs [44,45]. This was achieved in the present study, where the high solubility of NTZ within NLCs components was proved by the elevated value of drug entrapment (87.5%).

In the current work, the zeta potential of blank NLCs was −39 mV compared to −36 mV for NTZ-loaded NLC, which makes the formulated NLCs less prone to crumpling because of electrostatic repulsion. Moreover, the NTZ loading decreased the zeta potential of NLCs because it neutralizes some of the surface charges of the NLCs. The similar observation has been reported for lipid nanoparticles, probably arising from the interaction between the negatively charged NLCs components and the positively charged ionized secondary amine of NTZ [46]. Furthermore, storage at 4 °C maintained the physicochemical characteristics of NLCs throughout the study period with no fluctuations in particle size, PDI, and almost constant zeta potential. Also, the EE stayed unaffected for the complete study interval.

The in vitro NTZ release from NLCs depends on numerous factors, involving the composition of nanoparticle, release medium pH, temperature and drug solubility. The release limiting component is the solid lipid stearic acid that construct a sort of shell around the liquid lipid [47]. In the present work, the drug release rate was directly dependent on the drug concentration, which characterizes a first-order model, and this was reported elsewhere [48]. The NTZ release form NLCs appears to follow diffusion mechanism of drug release pattern suggesting the sustained release effect. Similarly, this diffusion mechanism was reported in other NLCs [49].

Estimation of the parasite burden highlights the infection severity and has the potential ability to evaluate the treatment efficiency [11]. Through the whole study, lower oocysts load was observed among immunocompetent mice rather than immunosuppressed ones, elucidating the additive role of immunity in fighting the parasite [9]. Throwing light on the used treatments, TMP-SMX revealed a marked gradual reduction in parasite load up to the 14^th^ day P.I. However, a decline in the percent reduction (% R) was evident on the 30^th^ day P.I. This can be clearly explained by the possibility of recurrence that may arise within one to three months despite of the treatment efficiency [9,20]. All over the experimental periods of the study, NTZ showed a lower %R compared to TMP-SMX. Correspondingly, other studies reported that NTZ is a safer treatment alternative that can be used when the patient suffers from intolerance to TMP-SMX, allergic to sulfa or in pregnant females. Nevertheless, it possesses a relative lower efficiency compared to TMP-SMX due to its low solubility [1,3,12]. Remarkably, NTZ-loaded NLCs showed the highest significant oocysts percent reduction as compared to all other treatments. This reduction exceeded 98% on the 14^th^ day P.I in both immunocompetent and immunosuppressed models with no evident parasite recurrence on the 30^th^ day. The loading of NTZ on NLCs added a great value in achieving a nano-metric sustained release formula along with increasing the drug solubility which remarkably improved the drug tissue permeability and efficiency. This coincides with other studies that proved the efficacy of nanoparticles in lowering the parasite burden compared to the conventional drugs [5,9,11].

Results of parasite load in the current study were in accordance with the ultrastructural study of oocysts collected from infected mice. Most of the examined treated oocysts exhibited loss of the spherical shape and smooth surface. Several disfigurements were noticed in the form of ridges, furrows, erosions, and/or ulcerations. However, mice treated with NTZ-loaded NLCs showed noteworthy mutilation with deep cavitations and appeared even rupturing. This may be attributed to the mechanism of action of NTZ in disturbing the respiratory cycle through inhibition of the pyruvate ferredoxin oxidoreductase enzyme [50]. A direct in vitro effect of NTZ on different life cycle stages of intestinal coccidia starting from sporozoite invasion till zygote formation and oocysts shedding was documented. Additionally, NTZ was reported to have a direct action on coccidian oocysts, reducing their viability which can be demonstrated by the ultrastructural study [51,52]. The effect of all used drugs on the ultrastructure of oocysts were more apparent in the immunocompetent mice. This may be explained by the synergistic effect of the drugs in addition to the intact immune response on the parasite [9]. The effect of NTZ on the outer surface membrane of different gastrointestinal and urogenital protozoa was reported by a previous study using SEM. The ultrastructural changes were in the form of deformities, cellular swelling and outer membrane irregularities [28]. In addition, results were synchronizing with other studies that reported the marked morphological changes of coccidian protozoa after different nano-treatments [9,11].

The study of histopathological alterations caused by cyclosporiasis in murine model is scarce and the persistence of intestinal inflammation even after parasite clearance presents an enduring pathology [53]. Yet, the present work confirmed the attained results by examining the histopathological changes resembled in intestinal villous length and degree of inflammation. The infected untreated immunocompetent control showed short edematous villi with moderate to severe inflammation where these changes were more evident by time. These findings agree with those described by other authors through examining intestinal biopsies [29]. Further villous shortening along with lower inflammatory response were observed in infected untreated immunosuppressed mice. This can be evidently linked to the immunosuppressive state that permits the parasite growth, multiplication and consequently the associated tissue damage [9]. All the used treatments improved the intestinal pathological picture. Meanwhile, NTZ-loaded NLCs produced the most prominent significant improvement almost imitating the normal uninfected control. Similar histopathological results were obtained by other authors in the treatment of different coccidian parasitic infections [54,55].

## Conclusion

In conclusion, the current study presented a bird’s eye view on the potent efficiency of the novel NTZ-loaded NLCs in treating cyclosporiasis without evident recurrence in both immunocompetent and immunosuppressed models. This in turn, affords an encouraging treatment platform based on the anti-parasitic and safe nature of the used formula.

## References

[1] Giangaspero A, Gasser RB. Human cyclosporiasis. Lancet Infect Dis. 2019 Jul;19(7):e226–e236. doi: 10.1016/S1473-3099(18)30789-8 30885589

[2] Almeria S, Cinar HN, Dubey JP. *Cyclospora cayetanensis* and cyclosporiasis: an update. Microorganisms. 2019 Sep 4;7(9):317. doi: 10.3390/microorganisms7090317 31487898 PMC6780905

[3] Li J, Cui Z, Qi M, Zhang L. Advances in cyclosporiasis diagnosis and therapeutic intervention. Front Cell Infect Microbiol. 2020 Feb 11;10:43. doi: 10.3389/fcimb.2020.00043 32117814 PMC7026454

[4] Satheeshkumar S, Ananthan S. Electron microscopy identification of microsporidia (*Enterocytozoon bieneusi*) and *Cyclospora cayetanensis* from stool samples of HIV infected patients. Indian J Med Microbiol. 2004 Apr-Jun;22(2):119–122. 17642709

[5] Hussein EM, Ahmed SA, Mokhtar AB, Elzagawy SM, Yahi SH, Hussein AM, et al. Antiprotozoal activity of magnesium oxide (MgO) nanoparticles against *Cyclospora cayetanensis* oocysts. Parasitol Int. 2018 Dec;67(6):666–674. doi: 10.1016/j.parint.2018.06.009 29933042

[6] Zerpa R, Uchima N, Huicho L. *Cyclospora cayetanensis* associated with watery diarrhoea in Peruvian patients. J Trop Med Hyg. 1995 Oct;98(5):325–329. 7563260

[7] Verdier RI, Fitzgerald DW, Johnson WD Jr, Pape JW. Trimethoprim-sulfamethoxazole compared with ciprofloxacin for treatment and prophylaxis of *Isospora belli* and *Cyclospora cayetanensis* infection in HIV-infected patients. A randomized, controlled trial. Ann Intern Med. 2000 Jun 6;132(11):885–888. doi: 10.7326/0003-4819-132-11-200006060-00006 10836915

[8] Guerrant RL, Van Gilder T, Steiner TS, Thielman NM, Slutsker L, Tauxe RV, et al. Infectious Diseases Society of America. Practice guidelines for the management of infectious diarrhea. Clin Infect Dis. 2001 Feb 1;32(3):331–351. doi: 10.1086/318514 11170940

[9] Gaafar MR, El-Zawawy LA, El-Temsahy MM, Shalaby TI, Hassan AY. Silver nanoparticles as a therapeutic agent in experimental cyclosporiasis. Exp Parasitol. 2019 Dec;207:107772. doi: 10.1016/j.exppara.2019.107772 31610183

[10] Tačić A, Nikolić V, Nikolić L, Savić I. Antimicrobial sulfonamide drugs. Adv technologies. 2017;6(1):58–71.

[11] Hagras NA, Mogahed NMFH, Sheta E, Darwish AA, El-Hawary MA, Hamed MT, et al. The powerful synergistic effect of spiramycin/propolis loaded chitosan/alginate nanoparticles on acute murine toxoplasmosis. PLoS Negl Trop Dis. 2022 Mar 16;16(3):e0010268. doi: 10.1371/journal.pntd.0010268 35294434 PMC8926208

[12] Bednarska M, Bajer A, Welc-Falęciak R, Pawełas A. *Cyclospora cayetanensis* infection in transplant traveller: a case report of outbreak. Parasit Vectors. 2015 Aug 7;8:411. doi: 10.1186/s13071-015-1026-8 26249024 PMC4528381

[13] Aljohani FS, Rezki N, Aouad MR, Elwakil BH, Hagar M, Sheta E, et al. Synthesis, characterization and nanoformulation of novel sulfonamide-1,2,3-triazole molecular conjugates as potent antiparasitic agents. Int J Mol Sci. 2022 Apr 11;23(8):4241. doi: 10.3390/ijms23084241 35457059 PMC9025934

[14] Hou X, Zaks T, Langer R, Dong Y. Lipid nanoparticles for mRNA delivery. Nat Rev Mater. 2021;6(12):1078–1094. doi: 10.1038/s41578-021-00358-0 34394960 PMC8353930

[15] Leong EWX, Ge R. Lipid nanoparticles as delivery vehicles for inhaled therapeutics. Biomedicines. 2022 Sep 2;10(9):2179. doi: 10.3390/biomedicines10092179 36140280 PMC9496059

[16] Beloqui A, Solinís MÁ, Rodríguez-Gascón A, Almeida AJ, Préat V. Nanostructured lipid carriers: Promising drug delivery systems for future clinics. Nanomedicine. 2016 Jan;12(1):143–161. doi: 10.1016/j.nano.2015.09.004 26410277

[17] Dhiman N, Awasthi R, Sharma B, Kharkwal H, Kulkarni GT. Lipid nanoparticles as carriers for bioactive delivery. Front Chem. 2021 Apr 23;9:580118. doi: 10.3389/fchem.2021.580118 33981670 PMC8107723

[18] Naseri N, Valizadeh H, Zakeri-Milani P. Solid lipid nanoparticles and nanostructured lipid carriers: structure, preparation and application. Adv Pharm Bull. 2015 Sep;5(3):305–13. doi: 10.15171/apb.2015.043 26504751 PMC4616893

[19] Asadpour M, Namazi F, Razavi SM, Nazifi S. Comparative efficacy of curcumin and paromomycin against *Cryptosporidium parvum* infection in a BALB/c model. Vet Parasitol. 2018 Jan 30;250:7–14. doi: 10.1016/j.vetpar.2017.12.008 29329627

[20] Garcia LS. Intestinal protozoa (coccidia), microsporidia and algae. In: Diagnostic Medical Parasitology. 6th ed. Washington, D.C.: ASM press; 2016. pp. 612–666.

[21] Smith HV, Paton CA, Mitambo MM, Girdwood RW. Sporulation of *Cyclospora sp*. oocysts. Appl Environ Microbiol. 1997 Apr;63(4):1631–2. 9097458 10.1128/aem.63.4.1631-1632.1997 PMC168455

[22] Shawky S, Makled S, Awaad A, Boraie N. Quercetin loaded cationic solid lipid nanoparticles in a mucoadhesive in situ gel-a novel intravesical therapy tackling bladder cancer. Pharmaceutics. 2022 Nov 20;14(11):2527. doi: 10.3390/pharmaceutics14112527 36432718 PMC9695231

[23] Makled S, Boraie N, Nafee N. Nanoparticle-mediated macrophage targeting-a new inhalation therapy tackling tuberculosis. Drug Deliv Transl Res. 2021 Jun;11(3):1037–1055. doi: 10.1007/s13346-020-00815-3 32617866

[24] Li X, Brasseur P, Agnamey P, Leméteil D, Favennec L, Ballet JJ, et al. Long-lasting anticryptosporidial activity of nitazoxanide in an immunosuppressed rat model. Folia Parasitol (Praha). 2003 Mar;50(1):19–22. doi: 10.14411/fp.2003.003 12735719

[25] Al-Mathal EM, Alsalem AM. Pomegranate (Punica granatum) peel is effective in a murine model of experimental *Cryptosporidium parvum*. Exp Parasitol. 2012 Jul;131(3):350–357. doi: 10.1016/j.exppara.2012.04.021 22580265

[26] Ma P, Soave R. Three-step stool examination for cryptosporidiosis in 10 homosexual men with protracted watery diarrhea. J Infect Dis. 1983 May;147(5):824–828. doi: 10.1093/infdis/147.5.824 6842020

[27] Clarke SC, McIntyre M. Modified detergent Ziehl-Neelsen technique for the staining of *Cyclospora cayetanensis*. J Clin Pathol. 1996 Jun;49(6):511–512. doi: 10.1136/jcp.49.6.511 8763270 PMC500546

[28] Cedillo-Rivera R, Chávez B, González-Robles A, Tapia A, Yépez-Mulia L. In vitro effect of nitazoxanide against *Entamoeba histolytica*, *Giardia intestinalis* and *Trichomonas vaginalis* trophozoites. J Eukaryot Microbiol. 2002 May-Jun;49(3):201–208. doi: 10.1111/j.1550-7408.2002.tb00523.x 12120985

[29] Mathison BA, Pritt BS. Cyclosporiasis-updates on clinical presentation, pathology, clinical diagnosis, and treatment. Microorganisms. 2021 Sep 2;9(9):1863. doi: 10.3390/microorganisms9091863 34576758 PMC8471761

[30] Ashour DS, Abou Rayia DM, Saad AE, El-Bakary RH. Nitazoxanide anthelmintic activity against the enteral and parenteral phases of trichinellosis in experimentally infected rats. Exp Parasitol. 2016 Nov;170:28–35. doi: 10.1016/j.exppara.2016.08.009 27585500

[31] Ley C. Flexible modelling in statistics: past, present and future. J Soc Fr Statistique. 2015;156(1):76–96.

[32] Meltzer E, Lachish T, Schwartz E. Treatment of giardiasis after nonresponse to nitroimidazole. Emerg Infect Dis. 2014 Oct;20(10):1742–1744. doi: 10.3201/eid2010.140073 25271363 PMC4193167

[33] Moawad H, Hgab M, Badawey M, Ashoush S, Ali A, Ibrahim S. Ameliorating effect of nitazoxanide loaded chitosan nanoparticles on intestinal dysplastic changes in immunosuppressed cryptosporidiosis murine model. J Egypt Soc Parasitol. 2022;52(3):509–518. doi: 10.21608/jesp.2022.278374

[34] Abdelhamed EF, Fawzy EM, Ahmed SM, Zalat RS, Rashed HE. Effect of nitazoxanide, artesunate loaded polymeric nano fiber and their combination on experimental cryptosporidiosis. Iran J Parasitol. 2019 Apr-Jun;14(2):240–249. 31543912 PMC6737357

[35] Ramadan HK, Hassan WA, Elossily NA, Ahmad AA, Mohamed AA, Abd-Elkader AS, et al. Evaluation of nitazoxanide treatment following triclabendazole failure in an outbreak of human fascioliasis in Upper Egypt. PLoS Negl Trop Dis. 2019 Sep 25;13(9):e0007779. doi: 10.1371/journal.pntd.0007779 31553716 PMC6779272

[36] Hagras NA, Allam AF, Farag HF, Osman MM, Shalaby TI, Mogahed NMFH, et al. Successful treatment of acute experimental toxoplasmosis by spiramycin-loaded chitosan nanoparticles. Exp Parasitol. 2019 Sep;204:107717. doi: 10.1016/j.exppara.2019.107717 31228418

[37] Round AN, Rigby NM, Garcia de la Torre A, Macierzanka A, Mills EN, Mackie AR. Lamellar structures of MUC2-rich mucin: a potential role in governing the barrier and lubricating functions of intestinal mucus. Biomacromolecules. 2012 Oct 8;13(10):3253–3261. doi: 10.1021/bm301024x 22978827

[38] Barua S, Mitragotri S. Challenges associated with penetration of nanoparticles across cell and tissue barriers: a review of current status and future prospects. Nano Today. 2014 Apr 1;9(2):223–243. doi: 10.1016/j.nantod.2014.04.008 25132862 PMC4129396

[39] Mohi-Eldin MM, Haridy MA, Hussein Khalil AM, Abdelnaeim K. Immunomodulatory and antiparasitic effects of garlic extract loaded on zinc oxide nanoparticles compared with pure garlic extract on *Eimeria stiedae*-infected rabbits. Benha Vet Med J. 2018 Sep 1;35(1):94–105. doi: 10.21608/bvmj.2018.38214

[40] Gelperina S, Kisich K, Iseman MD, Heifets L. The potential advantages of nanoparticle drug delivery systems in chemotherapy of tuberculosis. Am J Respir Crit Care Med. 2005 Dec 15;172(12):1487–1490. doi: 10.1164/rccm.200504-613PP 16151040 PMC2718451

[41] Darwish WM, Bayoumi NA, El-Kolaly MT. Laser-responsive liposome for selective tumor targeting of nitazoxanide nanoparticles. Eur J Pharm Sci. 2018 Jan 1;111:526–533. doi: 10.1016/j.ejps.2017.10.038 29097304

[42] Pinto EG, Barbosa LRS, Mortara RA, Tempone AG. Targeting intracellular *Leishmania (L*.*) infantum* with nitazoxanide entrapped into phosphatidylserine-nanoliposomes: An experimental study. Chem Biol Interact. 2020 Dec 1;332:109296. doi: 10.1016/j.cbi.2020.109296 33096056 PMC7573672

[43] Hassan MM, El-Rahman A, Mostafa E, El-Hamed A, Fakhry E, Abdel Fattah AS, et al. The impact of nitazoxanide loaded on solid lipid nanoparticles on experimental trichinellosis. Zagazig Univ Med J. 2021 Nov 1;27(6):1074–1084. doi: 10.21608/zumj.2019.16531.1480

[44] Hu FQ, Jiang SP, Du YZ, Yuan H, Ye YQ, Zeng S. Preparation and characterization of stearic acid nanostructured lipid carriers by solvent diffusion method in an aqueous system. Colloids Surf B Biointerfaces. 2005 Nov 10;45(3–4):167–173. doi: 10.1016/j.colsurfb.2005.08.005 16198092

[45] Tran TH, Ramasamy T, Truong DH, Choi HG, Yong CS, Kim JO. Preparation and characterization of fenofibrate-loaded nanostructured lipid carriers for oral bioavailability enhancement. AAPS PharmSciTech. 2014 Dec;15(6):1509–1515. doi: 10.1208/s12249-014-0175-y 25035071 PMC4245431

[46] Abbasalipourkabir R, Fallah M, Sedighi F, Maghsood AH, Javid S. Nanocapsulation of nitazoxanide in solid lipid nanoparticles as a new drug delivery system and in vitro release study. J Biol Sci. 2016;16(4):120–127. doi: 10.3923/jbs.2016.120.127

[47] Souto EB, Baldim I, Oliveira WP, Rao R, Yadav N, Gama FM, et al. SLN and NLC for topical, dermal, and transdermal drug delivery. Expert Opin Drug Deliv. 2020 Mar;17(3):357–377. doi: 10.1080/17425247.2020.1727883 32064958

[48] Wu H, Wang X, Zheng J, Zhang L, Li X, Yuan WE, et al. Propranolol-loaded mesoporous silica nanoparticles for treatment of infantile hemangiomas. Adv Healthc Mater. 2019 May;8(9):e1801261. doi: 10.1002/adhm.201801261 30838782

[49] Faiz S, Arshad S, Kamal Y, Imran S, Asim MH, Mahmood A, et al. Pioglitazone-loaded nanostructured lipid carriers: In-vitro and in-vivo evaluation for improved bioavailability. J Drug Deliver Sci Tech. 2023 Jan 1;79:104041. doi: 10.1016/j.jddst.2022.104041

[50] Broekhuysen J, Stockis A, Lins RL, De Graeve J, Rossignol JF. Nitazoxanide: pharmacokinetics and metabolism in man. Int J Clin Pharmacol Ther. 2000 Aug;38(8):387–394. doi: 10.5414/cpp38387 10984012

[51] Gargala G, Delaunay A, Li X, Brasseur P, Favennec L, Ballet JJ. Efficacy of nitazoxanide, tizoxanide and tizoxanide glucuronide against *Cryptosporidium parvum* development in sporozoite-infected HCT-8 enterocytic cells. J Antimicrob Chemother. 2000 Jul 1;46(1):57–60. doi: 10.1093/jac/46.1.57 10882689

[52] Sharma P, Sharma A, Sehgal R, Khurana S. Differential effect of paromomycin and nitazoxanide on clinical isolates of *Cryptosporidia* in vitro. Int J Appl Biol and Pharm Tech. 2014 Nov; 5(4):134–146.

[53] Bartelt LA, Bolick DT, Kolling GL, Roche JK, Zaenker EI, Lara AM, et al. Cryptosporidium Priming is more effective than vaccine for protection against cryptosporidiosis in a murine protein malnutrition model. PLoS Negl Trop Dis. 2016 Jul 28;10(7):e0004820. doi: 10.1371/journal.pntd.0004820 27467505 PMC4965189

[54] Farid A, Tawfik A, Elsioufy B, Safwat G. In vitro and in vivo anti-Cryptosporidium and anti-inflammatory effects of Aloe vera gel in dexamethasone immunosuppressed mice. Int J Parasitol Drugs Drug Resist. 2021 Dec;17:156–167. doi: 10.1016/j.ijpddr.2021.09.002 34637982 PMC8503859

[55] Allam AF, Hagras NA, Farag HF, Osman MM, Shalaby TI, Kazem AH, et al. Remarkable histopathological improvement of experimental toxoplasmosis after receiving spiramycin-chitosan nanoparticles formulation. J Parasit Dis. 2022 Mar;46(1):166–177. doi: 10.1007/s12639-021-01431-9 35299902 PMC8901813

